# Supplementary material for: Understanding climate-sensitive diseases in Bangladesh using systematic review and government data repository
Source: PLoS One. 2025 Mar 19;20(3):e0313031. doi: 10.1371/journal.pone.0313031 (PMC11922245; doi:10.1371/journal.pone.0313031)
Supplement: S1 File — (DOCX) [file pone.0313031.s001.docx]

**Addressing Data Challenges for Understanding Climate-Sensitive Diseases in Bangladesh: Evidence from Systematic Review and Government Data Repository**

**Supplementary Table 1:** Search strategy of Medline database (January 2000 to December 2022)

| **#** | **Searches** | **Results** |
| --- | --- | --- |
| 1 | (climate change or adaptation or vulnerable community or vulnerabilities).mp. [mp=title, book title, abstract, original title, name of substance word, subject heading word, floating sub-heading word, keyword heading word, organism supplementary concept word, protocol supplementary concept word, rare disease supplementary concept word, unique identifier, synonyms] | 419273 |
| 2 | Bangladesh/ | 13870 |
| 3 | 1 and 2 | 308 |

**Supplementary Table 2:** Search strategy of Scopus database (January 2000 to December 2022)

| Searches | Results |
| --- | --- |
| TITLE-ABS-KEY ( ( climate  AND change  OR  adaptation  OR  vulnerable  AND community  OR  vulnerabilities )  AND  Bangladesh ) | 18 |

**Supplementary Table 3:** Search strategy of Embase database (January 2000 to December 2022)

| **#** | **Searches** | **Results** |
| --- | --- | --- |
| 1 | (climate change or adaptation or vulnerable community or vulnerabilities).mp. [mp=title, abstract, heading word, drug trade name, original title, device manufacturer, drug manufacturer, device trade name, keyword heading word, floating subheading word, candidate term word] | 412058 |
| 2 | Bangladesh/ | 20659 |
| 3 | 1 and 2 | 311 |

**Supplementary Table 4:** Search strategy of Maternity & Infant Care Database (January 2000 to December 2022)

| **#** | **Searches** | **Results** |
| --- | --- | --- |
| 1 | (climate change or adaptation or vulnerable community or vulnerabilities).mp. [mp=abstract, heading word, title] | 3607 |
| 2 | bangladesh.mp. [mp=abstract, heading word, title] | 1008 |
| 3 | 1 and 2 | 8 |

**Supplementary Table 5:** Search strategy of PsychoINFO Database (January 2000 to December 2022)

| **#** | **Searches** | **Results** |
| --- | --- | --- |
| 1 | (climate change or adaptation or vulnerable community or vulnerabilities).mp. [mp=title, abstract, heading word, drug trade name, original title, device manufacturer, drug manufacturer, device trade name, keyword heading word, floating subheading word, candidate term word] | 1808 |
| 2 | Bangladesh/ | 789 |
| 3 | 1 and 2 | 698 |
| 4 | limit 3 to yr="2000 -Current" | 385 |

**Supplementary Table 6:** Search strategy of Web of Science Database (January 2000 to December 2022)

| Searches | Results |
| --- | --- |
| climate change or adaptation or vulnerable community or vulnerabilities (All Fields) and ****Bangladesh**** (All Fields) and >1999 | 390 |

**Supplemental Table 7.** Newcastle-Ottawa scale assessment of study quality for **cross-sectional study**

| Author | Selection | | | | Comparability | Outcome | | Study quality |
| --- | --- | --- | --- | --- | --- | --- | --- | --- |
|  | 1 | 2 | 3 | 4 | 5 | 6 | 7 |  |
|  | Representativeness of the sample | Sample size | Ascertainment of exposure | Non-respondents | The subjects in different outcome groups are comparable, based on the study design or analysis. Confounding factors are controlled. | Assessment of outcome | Statistical test is appropriate |  |
| (Banu, Hu et al. 2014) | * | * | * | * | * | * | * | 7 |
| (Hsan, Hossain et al. 2019) | * | * |  |  | * | * | * | 5 |
| (Rahman, Sharker et al. 2020) |  | * | * | * | * | * | * | 6 |
| (Matsuda, Ishimura et al. 2008) | * | * |  |  | * |  | * | 4 |
| (Sharker, Rheman et al. 2009) | * | * |  |  | * |  | * | 4 |
| (Wu, Yunus et al. 2014) |  |  | * | * | * | * | * | 5 |
| (Molla, Mollah et al. 2014) | * | * | * | * | * | * | * | 7 |
| (Molla, Mollah et al. 2014) | * | * | * | * | * | * | * | 7 |
| (Hashizume, Armstrong et al. 2007) | * | * | * | * | * | * | * | 7 |
| (Emeto, Adegboye et al. 2020) | * | * | * |  | * | * | * | 6 |
| (Hossain, Tong et al. 2020) | * | * | * | * | * | * | * | 7 |
| (Chowdhury, Ibrahim et al. 2018) |  | * | * | * | * | * | * | 6 |
| (Dewan, Hashizume et al. 2016) |  | * |  |  | * |  |  | 2 |
| (Nurhamim 2020) | * | * |  |  | * |  |  | 3 |
| (Kabir, Rahman et al. 2016) | * | * | * | * | * | * | * | 7 |
| (Ashrafuzzaman and Furini 2019) |  |  | * |  | * | * |  | 3 |
| (Haque, Parr et al. 2019) | * | * | * | * | * | * | * | 7 |
| (Hossain, Shi et al. 2022) |  |  | * | * | * | * | * | 5 |
| (Talukder, Rutherford et al. 2016) |  | * | * | * | * | * | * | 6 |
| (Chakraborty, Khan et al. 2019) | * | * | * | * | * | * | * | 7 |
| (Rasheed, Siddique et al. 2016) |  | * | * | * | * | * | * | 6 |
| (Chen, Atiqul Haq et al. 2021 | * | * | * |  | * | * | * | 6 |
| (Kabir 2018) | * |  | * |  | * | * | * | 5 |
| (Ashraf and Faruk 2018) |  |  | * | * | * | * | * | 5 |
| (Kruger, Lorah et al. 2022) | * | * |  | * |  |  | * | 4 |
| (Haque, Hashizume et al. 2010) | * | * | * | * | * | * | * | 7 |

**Supplemental Table 8.** Newcastle-Ottawa scale assessment of study quality for **case-control study**

| Author | Selection | | | | Comparability | | Exposure | | | Study quality |
| --- | --- | --- | --- | --- | --- | --- | --- | --- | --- | --- |
|  | 1 | 2 | 3 | 4 | 5A | 5B | 6 | 7 | 8 |  |
|  | Is the case definition adequate? | Representativeness of the cases | Selection of controls | Definition of controls | Case-control comparable on basis of age | Case-control comparable on other factor(s) | Ascertainment of exposure | Same method of ascertainment for cases and control | Non-response rate |  |
| (Scheelbeek, Khan et al. 2016) | * | * | * | * |  | * |  | * |  | 6 |
| (Wu, Yunus et al. 2018) | * | * | * | * |  | * |  |  |  | 5 |

**Supplemental Table 9.** Newcastle-Ottawa scale assessment of study quality for **Cohort study**

| Author | Selection | | | | Comparability | Study quality | | | Study quality |
| --- | --- | --- | --- | --- | --- | --- | --- | --- | --- |
|  | 1 | 2 | 3 | 4 | 5 | 6 | 7 | 8 |  |
|  | Representativeness of the Exposed Cohort | Selection of the Non-Exposed Cohort | Ascertainment of Exposure | Demonstration that the current outcome of interest was not present at start of study | Comparability of Cohorts on the Basis of the Design or Analysis | Assessment of Outcome | Was the Follow-Up Long Enough for Outcomes to Occur? | Adequacy of follow up of cohorts. |  |
| (Grembi, Nguyen et al. 2022) | * | * | * | * | * | * | * | * | 8 |
| (Scheelbeek, Chowdhury et al. 2017) | * | * | * |  | * | * | * | * | 7 |

**Supplemental Table 10.** Newcastle-Ottawa scale assessment of study quality for **Qualitative study**

| Author | Selection | | | | Confounder | Exposure | | | Study quality |
| --- | --- | --- | --- | --- | --- | --- | --- | --- | --- |
|  | 1 | 2 | 3 | 4 | 5 | 6 | 7 | 8 |  |
|  | Is the case definition adequate? | Representativeness of the cases | Selections of controls | Definitions of Controls | Comparability of cases and controls on the basis of design or analysis | Ascertainment of exposure | Same method of ascertainment for cases and controls | Non-responsrate |  |
| (Abdullah, Dalal et al. 2019) | * |  | * | * | * | * | * | * | 7 |
| (Nayna Schwerdtle, Baernighausen et al. 2021) | * | * | * | * | * | * | * |  | 7 |
| (Rashid and Michaud 2000) | * | * | * | * | * | * | * | * | 8 |

**Supplemental Table 11.** Newcastle-Ottawa scale assessment of study quality for **Randomized control trial**

| Author | Selection | | | | Comparability | | Exposure | | | Study quality |
| --- | --- | --- | --- | --- | --- | --- | --- | --- | --- | --- |
|  | 1 | 2 | 3 | 4 | 5 | 6 | 7 | 8 | 9 |  |
|  | Is the case definition adequate? | Representativeness of the cases | Selection of Controls | Definition of Controls | study controls for (Select the most important factor | study controls for any additional factor (This criteria could be modified to indicate specific control for a second important factor.) | Ascertainment of exposure | Same method of ascertainment for cases and controls | Non-Response rate |  |
| (Nguyen, Grembi et al. 2022) | * | * | * | * | * | * | * | * | * | 9 |

**Supplementary Table 12: Characteristics of the study included in systematic review that reported climate sensitive diseases**

| Author’s, Year of publication | Outcome | Type of Study Design | Study location | Year of study | Focus |
| --- | --- | --- | --- | --- | --- |
| Communicable Diseases (CDs) | | | | | |
| (Banu, Hu et al. 2014) | Dengue | Cross sectional | Dhaka | 2000-2010 | This paper evaluates the effects of weather variability on dengue transmission in Dhaka, Bangladesh, and predicts the future impact of climate change on dengue. The study indicates a positive correlation between temperature and humidity with dengue transmission, forecasting a rise in dengue cases in Dhaka due to climate change. |
| (Hsan, Hossain et al. 2019) | Dengue | Cross sectional | Bangladesh | 2018-2019 | This paper highlights the need to enhance prevention and control strategies for dengue in Bangladesh, emphasizing community-based measures to reduce mosquito breeding sites, early detection tools, and training for clinicians. It also addresses the impact of climate change, urbanization, and healthcare systems on dengue outbreaks in the country. |
| (Rahman, Sharker et al. 2020) | Dengue | Cross sectional | Dhaka | 2005-2009 | This paper investigates the influence of climatic variability on the occurrence of clinical dengue requiring hospitalization in Zone-5, a high incidence area of Dhaka City Corporation (DCC), Bangladesh. The study found that the number of rainy days per month is significantly associated with dengue incidence in the subsequent month. |
| (Kruger, Lorah et al. 2022) | Cholera | Case Control | Bangladesh | 2015-2050 | This paper models the likelihood of cholera outbreaks in Bangladesh using environmental variables, such as rainfall, temperature, and water pH levels. Risk maps for cholera infection in 2015 and 2050 are generated, providing guidance for public health policy to prevent future outbreaks. |
| (Matsuda, Ishimura et al. 2008) | Cholera | Cross sectional | Dhaka | 1983-2002 | This article predicts cholera epidemics using climate data, utilizing autoregression analysis to develop a model that forecasts monthly patient numbers based on previous climate variables. |
| (Sharker, Rheman et al. 2009) | Cholera | Cross sectional | Matlab | 1989-2005 | This paper examines the influence of climate variability on cholera seasonality and transmission in Bangladesh, identifying a synergistic effect of temperature and sunshine hours on the occurrence of cholera, with the highest incidence observed when both factors are high. |
| (Wu, Yunus et al. 2018) | Cholera | Cross sectional | Matlab | 1983-2009 | This paper examines the association between heatwaves and cholera outbreaks in a cholera endemic area of rural Bangladesh. The study found that heatwaves increase the risk of cholera, and this relationship is modified by rainfall and tree cover. |
| (Wu, Yunus et al. 2014) | Diarrhoea | Cross sectional | Matlab | 2000-2006 | This study examines the impact of meteorological factors, particularly extreme weather events, on the prevalence of childhood diarrhoeal disease in rural areas of Bangladesh. The results show that both the intensity and frequency of extreme weather events have significant effects on childhood diarrhoea. |
| (Molla, Mollah et al. 2014) | Diarrhoea And Asthma | Cross sectional | Dhaka | 2012 | This article examines the health status of climate refugees versus non-climate refugees in low-income areas of Dhaka, focusing on water supply, sanitation, and indoor air contaminants. The study highlights their impact on reducing the burden of disease, such as DALYs lost from diarrhoea and asthma. |
| (Molla, Mollah et al. 2014) | Diarrhoea And Asthma | Cross sectional | Dhaka | 2012 | This paper evaluates the impact of climate change on vulnerable communities in Dhaka by comparing DALYs lost from diarrhoea and asthma in climate and non-climate refugee groups under 5 years old. The findings emphasize the importance of prioritizing policy interventions for climate refugees. |
| (Nguyen, Grembi et al. 2022) | Diarrhoea | RCT | Gazipur, Kishoreganj, Mymensingh, Tangail | 2012-2013 | This paper examines the impact of low-cost WASH interventions on diarrhoea and their effectiveness in a changing climate. The study indicates that WASH interventions are most effective under higher precipitation, and their effectiveness may improve without sustainable development in the face of climate change. |
| (Grembi, Nguyen et al. 2022) | Diarrhoea | Cohort | Gazipur, Kishoreganj, Mymensingh, Tangail | 2012-2013 | This paper explores the correlation between climate and diarrhoeal diseases caused by enteric pathogens in rural Bangladesh. The study reveals that increased temperatures and precipitation are linked to a higher prevalence of diarrhoea and multiple entomopathogens, with expected future climate changes likely to increase the incidence of clinical illness. |
| (Hashizume, Armstrong et al. 2007) | Non-Cholera Diarrhoea | Cross sectional | Patients visiting  (ICDDR, B), Dhaka | 1996-2002 | This paper analyzes the impact of rainfall and temperature on non-cholera diarrhoea cases in Dhaka, Bangladesh. The study discovers that both above and below a threshold level of rainfall in the preceding weeks increase the number of cases, while temperature has a stronger effect on individuals with lower socio-economic and sanitation status. |
| (Haque, Hashizume et al. 2010) | Malaria | Cohort | Rangamati district hospital | 1989-2008 | This paper examines the correlation between climatic factors and malaria cases in the Chittagong Hill Tracts district of Bangladesh over 20 years. The study shows no evidence for any association between malaria cases and temperature, rainfall, and humidity, but a low NDVI is linked to an increase in the number of malaria cases. |
| (Emeto, Adegboye et al. 2020) | Malaria | Cross sectional | UHC,Rajasthali, Rangamati | 2000-2009 | This paper explores the connection between climate variability and malaria incidence in Rangamati, Bangladesh. The study discovers a significant positive correlation between temperature and rainfall with malaria occurrence, with two peaks observed at 19 °C and 24.5°C for temperature and at 86 mm and 284 mm for rainfall. |
| (Hossain, Tong et al. 2020) | Pneumonia | Cross sectional | Matlab | 2012-2016 | This study explores the connection between climate variability, patient characteristics, and hospital stay duration for childhood pneumonia in rural Bangladesh. The findings indicate that climate variation plays a significant role in hospital stay duration, emphasizing the need for policymakers to develop effective strategies for disease management and prevention. |
| (Chowdhury, Ibrahim et al. 2018) | Malaria, Diarrheal Disease, Enteric Fever, Encephalitis, Pneumonia, And Bacterial Meningitis. | Cross sectional | Bangladesh | 2008-2012 | This observational study investigates the correlation between temperature, humidity, rainfall, and six climate-sensitive infectious diseases in adults in north-eastern Bangladesh. The results suggest a connection between weather patterns and disease incidence, providing vital baseline data for future prospective studies. |
| (Dewan, Hashizume et al. 2016) | Kala-Azar | Cross sectional | Mymensingh, Tangail, Gazipur, Pabna, Jamalpur, Khulna, Panchagar, Rajshahi, and Sirajganj. | 2008-2014 | This paper examines the incidence of visceral leishmaniasis (kala-azar) in Bangladesh from historical and current perspectives. It also explores the future vulnerability of the country and its inhabitants under conditions of environmental change. |
| (Nurhamim 2020) | Skin Infection, Pneumonia, Respiratory Infection, Mosquito-Borne Illnesses, Hepatitis A Or E Virus Infection. | Cross sectional | Bangladesh | 1985-2019 | This study investigates flood disasters in Bangladesh, examining their socioeconomic and public health impacts. Analysis of quantitative data from multiple sources reveals floods as the most prevalent natural hazard, leading to considerable economic and public health consequences. |
| (Kabir, Rahman et al. 2016) | Dengue, Malaria, Diarrhea, And Pneumonia | Cross sectional | Bagerhat, Barguna, Cox’s Bazar, Faridpur, Khulna, Satkhira, and Sirajganj | 2012 | This paper discusses the potential impact of climate change on health in Bangladesh. It presents findings from a survey conducted among vulnerable communities in seven districts of Bangladesh, highlighting the high incidence of climate-sensitive diseases and the need for community-based adaptation strategies for health. |
| (Ashrafuzzaman and Furini 2019) | Dysentery, Skin Diseases and Diarrhea | Cross sectional | Shyamnagar Upazila | 2018 | This paper analyzes the impacts of climate change on public health, focusing on coastal communities in the area of influence of Sundarbans, located in the southwestern coastal region of Bangladesh. It discusses the major challenges faced by different actors in dealing with health problems caused by climate change. |
| (Haque, Parr et al. 2019) | Fever, Diarrhea, Jaundice, Typhoid, Acute Respiratory Infections and Gastrointestinal Diseases | Cross sectional | North-western mainland region of Bangladesh | 2017 | This paper analyzes the healthcare-seeking behavior of parents for their children in climate displacement-prone areas compared to non-prone areas in Bangladesh. The study shows that climate-related displacement influences healthcare-seeking behaviors and recommends policy interventions to enhance child health in these areas. |
| (Hossain, Shi et al. 2022) | Skin Diseases and Diarrhea | Cross sectional | Gaibandha | 2018-2019 | This paper examines the impact of climate change on the health of riverine island (char) dwellers in Bangladesh and their adaptation strategies. The study reveals flooding, riverbank erosion, drought, and disease outbreaks as the most significant climate change indicators perceived by the char inhabitants, causing various health-related issues throughout the year. |
| Non-Communicable Diseases (NCDs) | | | | | |
| (Talukder, Rutherford et al. 2016) | Hypertension, Cardiovascular Diseases, Kidney Diseases, Malnourished | Cross sectional | Koyra, (Khulna) | 2014 | This research examines the association between drinking water salinity and blood pressure in young adults in coastal Bangladesh. The study found that elevated salinity in drinking water is associated with higher blood pressure in young coastal populations. |
| (Chakraborty, Khan et al. 2019) | Cardiovascular, Diarrhea, Abdominal pain, Gastric ulcer, Dysentery, Skin Diseases, Typhoid | Cross sectional | Mathbaria ,Zianagar , Mongla | 2016-2017 | This paper investigates the association between high water salinity exposure and hospital visits for various health effects in coastal sub-districts of Bangladesh. The study found that high water salinity is significantly associated with cardiovascular diseases, diarrhea, and abdominal pain-related hospital visits. |
| (Scheelbeek, Chowdhury et al. 2017) | High blood pressure, Hypertension | Cohort | Dacope, Batiaghata, Paikghaccha | 2013-2014 | This paper investigates the effects of drinking-water sodium (DWS) on blood pressure (BP) in coastal populations in Bangladesh. The study found that DWS is an important source of daily sodium intake in salinity-affected areas and is a risk factor for hypertension. |
| (Scheelbeek, Khan et al. 2016) | High blood pressure, Hypertension, Cardiovascular Diseases | Case Control | Dacope, Khulna | 2009-2011 | High sodium in drinking water sources in coastal Bangladesh is associated with increased blood pressure in healthy pregnant women. Improved drinking water quality and considering environmental exposures are crucial in public health research. |
| (Rasheed, Siddique et al. 2016) | Increase blood pressure, Eclampsia, Hypertension, Cardiovascular Diseases, Heart attac, Stroke, Kidney Disease, Abdominal Cancer, Reduce breast milk production | Cross sectional | Chakaria | 2011 | This paper examines salt consumption in coastal Bangladesh, where climate change-induced saline intrusion in water has endangered 20 million people with hypertension. It explores beliefs, perceptions, and practices related to dietary salt sources, specifically among the at-risk coastal population facing environmental salinity. |
| Maternal Health Issues | | | | | |
| (Rashid and Michaud 2000) | Gota [Boils] And Chulkani [Itchy Rash], Perineal Rashes, Cramps and Urinary-Tract Infections, Fever, Diarrhea and Jaundice | Qualitative | Manikganj, Dhaka | 1998 | This paper discusses the experiences of adolescent girls during the 1998 floods in Bangladesh, focusing on the impact of cultural norms related to honor, shame, purity, and pollution. The floods made it difficult for girls to maintain their privacy and follow social norms, leading to mental and sexual harassment and health issues. |
| (Chen, Atiqul Haq et al. 2021) | Decrease Fertility | Cross sectional | Bangladesh | 1966-2015 | This paper examines the direct and indirect effects of climate change on fertility in Bangladesh through its impact on food production. The study uses structural equation modelling and time-series data analysis to show that climate change parameters and crop production are non-negligible factors in the fertility decline in Bangladesh. |
| (Abdullah, Dalal et al. 2019) | Malnutrition And Anemia, Urinary Tract Infections. | Qualitative | Khaliajhuri (Netrakona) | 2015 | This study examines the impact of floods on maternal health and the challenges faced by rural communities in providing care during such disasters. It emphasizes the need for pre-disaster preparation and increased awareness among local health providers to address maternal health during disasters. |
| Mental Health Issues | | | | | |
| (Kabir 2018) | Depression, Frustration, And Suicide Tendency | Cross sectional | Chittagong,  Cox’s Bazar, Rangamati,  Bandarban ,Khagrachhar | 2015-2016 | This paper examines the adverse effects of climate change on the mental health and well-being of the Hill-Tracts region's residents in Bangladesh. In-depth interviews with community members and health professionals reveal climate change as an additional psychological stressor for the region's inhabitants. |
| CDs & NCDs | | | | | |
| (Nayna Schwerdtle, Baernighausen et al. 2021) | Fever, Diarrhoea , Cough, Psychological Trauma, Body Aches | Qualitative | Bhola slum, Dhaka | 2019 | This paper explores the experiences of migrants who moved from rural Bhola, southern Bangladesh to an urban slum in Dhaka, Bangladesh, in the context of climate change. The study identifies two overarching themes: "A risk exchange: Exchanging climate change and health risks at origin and destination" and "A changing health and healthcare environment." |
| (Ashraf and Faruk 2018) | Diarrhea And Cholera, Sweating, Feeling Thirsty, Discomfort, Headache, Stomach Aches, Prickly-Heat, Getting Easily Irritated, Feeling Sluggish, Weakness and Dehydration, Cold and Fever, Irritation in Skin, Loss of Concentration | Cross sectional | Dhaka | 2012-2016 | This paper explores the perceptions and experiences of children in Dhaka, Bangladesh, regarding the impacts of increased heat and heavy precipitation due to climate change. The study aims to identify the vulnerabilities of children and their coping strategies to inform urban development and planning policies for the city. |

**Supplementary Table 13**. Climate sensitive disease mapping following international disease classification system 10 and its year-wise reported cases during the year 2017-2022.

| **Disease** | **2017** | | **2018** | | **2019** | | **2020** | | **2021** | | **2022** | | **Total** | |
| --- | --- | --- | --- | --- | --- | --- | --- | --- | --- | --- | --- | --- | --- | --- |
|  | **n** | **%** | **n** | **%** | **n** | **%** | **n** | **%** | **n** | **%** | **n** | **%** | **n** | **%** |
| A00 Cholera | 15321 | 4.27 | 7463 | 1.38 | 5196 | 0.81 | - | 0.00 | 6542 | 1.23 | 7540 | 1.28 | 42062 | 1.47 |
| A000 Cholera due to Vibrio cholerae 01, biovar cholerae | 3651 | 1.02 | 1316 | 0.24 | 860 | 0.13 | - | 0.00 | 1581 | 0.30 | 1045 | 0.18 | 8453 | 0.30 |
| A001 Cholera due to Vibrio cholerae 01, biovar eltor | 544 | 0.15 | 242 | 0.04 | 164 | 0.03 | - | 0.00 | 203 | 0.04 | 241 | 0.04 | 1394 | 0.05 |
| A009 Cholera, unspecified | 4983 | 1.39 | 9013 | 1.67 | 6246 | 0.97 | - | 0.00 | 8620 | 1.62 | 6068 | 1.03 | 34930 | 1.22 |
| A01 Typhoid and paratyphoid fevers | 4411 | 1.23 | 3359 | 0.62 | 4434 | 0.69 | - | 0.00 | 2960 | 0.56 | 4846 | 0.82 | 20010 | 0.70 |
| A010 Typhoid fever | 12565 | 3.50 | 17589 | 3.25 | 18185 | 2.82 | - | 0.00 | 8132 | 1.53 | 10546 | 1.79 | 67017 | 2.34 |
| A011 Paratyphoid fever A | 1528 | 0.43 | 945 | 0.17 | 312 | 0.05 | - | 0.00 | 145 | 0.03 | 174 | 0.03 | 3104 | 0.11 |
| A012 Paratyphoid fever B | 534 | 0.15 | 178 | 0.03 | 48 | 0.01 | - | 0.00 | 66 | 0.01 | 68 | 0.01 | 894 | 0.03 |
| A013 Paratyphoid fever C | 167 | 0.05 | 73 | 0.01 | 25 | 0.00 | - | 0.00 | 118 | 0.02 | 40 | 0.01 | 423 | 0.01 |
| A014 Paratyphoid fever, unspecified | 527 | 0.15 | 382 | 0.07 | 455 | 0.07 | - | 0.00 | 660 | 0.12 | 196 | 0.03 | 2220 | 0.08 |
| A03 Shigellosis | 90 | 0.03 | 67 | 0.01 | 55 | 0.01 | - | 0.00 | 140 | 0.03 | 175 | 0.03 | 527 | 0.02 |
| A030 Shigellosis due to Shigella dysenteriae | 108 | 0.03 | 160 | 0.03 | 198 | 0.03 | - | 0.00 | 66 | 0.01 | 65 | 0.01 | 597 | 0.02 |
| A031 Shigellosis due to Shigella flexneri | 30 | 0.01 | 71 | 0.01 | 8 | 0.00 | - | 0.00 | 31 | 0.01 | 13 | 0.00 | 153 | 0.01 |
| A032 Shigellosis due to Shigella boydii | 23 | 0.01 | 15 | 0.00 | 15 | 0.00 | - | 0.00 | 17 | 0.00 | 16 | 0.00 | 86 | 0.00 |
| A033 Shigellosis due to Shigella sonnei | 27 | 0.01 | 13 | 0.00 | 11 | 0.00 | - | 0.00 | 23 | 0.00 | 26 | 0.00 | 100 | 0.00 |
| A038 Other shigellosis | 893 | 0.25 | 166 | 0.03 | 101 | 0.02 | - | 0.00 | 43 | 0.01 | 30 | 0.01 | 1233 | 0.04 |
| A039 Shigellosis, unspecified | 68 | 0.02 | 55 | 0.01 | 11 | 0.00 | - | 0.00 | 44 | 0.01 | 18 | 0.00 | 196 | 0.01 |
| A05 Other bacterial foodborne intoxications | 141 | 0.04 | 148 | 0.03 | 191 | 0.03 | - | 0.00 | 122 | 0.02 | 84 | 0.01 | 686 | 0.02 |
| A050 Foodborne staphylococcal intoxication | 24 | 0.01 | 47 | 0.01 | 47 | 0.01 | - | 0.00 | 31 | 0.01 | 29 | 0.00 | 178 | 0.01 |
| A051 Botulism |  | 0.00 | 8 | 0.00 | 9 | 0.00 | - | 0.00 | 8 | 0.00 | 4 | 0.00 | 29 | 0.00 |
| A052 Foodborne Clostridium perfringens [Clostridium welchii] intoxication | 8 | 0.00 | 18 | 0.00 | 11 | 0.00 | - | 0.00 | 16 | 0.00 | 44 | 0.01 | 97 | 0.00 |
| A053 Foodborne Vibrio parahaemolyticus intoxication | 6 | 0.00 | 18 | 0.00 | 9 | 0.00 | - | 0.00 | 13 | 0.00 | 14 | 0.00 | 60 | 0.00 |
| A054 Foodborne Bacillus cereus intoxication | 7 | 0.00 | 17 | 0.00 | 12 | 0.00 | - | 0.00 | 11 | 0.00 | 42 | 0.01 | 89 | 0.00 |
| A058 Other specified bacterial foodborne intoxications | 26 | 0.01 | 14 | 0.00 | 25 | 0.00 | - | 0.00 | 12 | 0.00 | 25 | 0.00 | 102 | 0.00 |
| A059 Bacterial foodborne intoxication, unspecified | 122 | 0.03 | 234 | 0.04 | 213 | 0.03 | - | 0.00 | 213 | 0.04 | 104 | 0.02 | 886 | 0.03 |
| A06 Amoebiasis | 85 | 0.02 | 110 | 0.02 | 53 | 0.01 | - | 0.00 | 97 | 0.02 | 89 | 0.02 | 434 | 0.02 |
| A060 Acute amoebic dysentery | 852 | 0.24 | 1258 | 0.23 | 1011 | 0.16 | - | 0.00 | 684 | 0.13 | 741 | 0.13 | 4546 | 0.16 |
| A061 Chronic intestinal amoebiasis | 14 | 0.00 | 28 | 0.01 | 26 | 0.00 | - | 0.00 | 11 | 0.00 | 79 | 0.01 | 158 | 0.01 |
| A062 Amoebic nondysenteric colitis | 10 | 0.00 | 24 | 0.00 | 24 | 0.00 | - | 0.00 | 20 | 0.00 | 36 | 0.01 | 114 | 0.00 |
| A063 Amoeboma of intestine | 11 | 0.00 | 29 | 0.01 | 87 | 0.01 | - | 0.00 | 8 | 0.00 | 20 | 0.00 | 155 | 0.01 |
| A064 Amoebic liver abscess | 106 | 0.03 | 334 | 0.06 | 183 | 0.03 | - | 0.00 | 126 | 0.02 | 308 | 0.05 | 1057 | 0.04 |
| A065 Amoebic lung abscess | 60 | 0.02 | 155 | 0.03 | 102 | 0.02 | - | 0.00 | 69 | 0.01 | 190 | 0.03 | 576 | 0.02 |
| A066 Amoebic brain abscess | 22 | 0.01 | 38 | 0.01 | 20 | 0.00 | - | 0.00 | 34 | 0.01 | 129 | 0.02 | 243 | 0.01 |
| A067 Cutaneous amoebiasis | 10 | 0.00 | 32 | 0.01 | 9 | 0.00 | - | 0.00 | 7 | 0.00 | 18 | 0.00 | 76 | 0.00 |
| A068 Amoebic infection of other sites | 15 | 0.00 | 25 | 0.00 | 29 | 0.00 | - | 0.00 | 30 | 0.01 | 20 | 0.00 | 119 | 0.00 |
| A069 Amoebiasis, unspecified | 8 | 0.00 | 18 | 0.00 | 37 | 0.01 | - | 0.00 | 20 | 0.00 | 16 | 0.00 | 99 | 0.00 |
| A08 Viral and other specified intestinal infections | 279 | 0.08 | 1282 | 0.24 | 1485 | 0.23 | - | 0.00 | 48 | 0.01 | 272 | 0.05 | 3366 | 0.12 |
| A081 Acute gastroenteropathy due to Norwalk agent | 700 | 0.20 | 2090 | 0.39 | 2044 | 0.32 | - | 0.00 | 931 | 0.18 | 3462 | 0.59 | 9227 | 0.32 |
| A082 Adenoviral enteritis | 100 | 0.03 | 155 | 0.03 | 177 | 0.03 | - | 0.00 | 29 | 0.01 | 68 | 0.01 | 529 | 0.02 |
| A083 Other viral enteritis | 111 | 0.03 | 706 | 0.13 | 331 | 0.05 | - | 0.00 | 85 | 0.02 | 43 | 0.01 | 1276 | 0.04 |
| A084 Viral intestinal infection, unspecified | 29 | 0.01 | 43 | 0.01 | 61 | 0.01 | - | 0.00 | 135 | 0.03 | 21 | 0.00 | 289 | 0.01 |
| A085 Other specified intestinal infections | 414 | 0.12 | 42 | 0.01 | 51 | 0.01 | - | 0.00 | 43 | 0.01 | 49 | 0.01 | 599 | 0.02 |
| A09 Diarrhoea and gastroenteritis of presumed infectious origin | 68917 | 19.20 | 164950 | 30.49 | 188582 | 29.26 | - | 0.00 | 183838 | 34.65 | 144182 | 24.50 | 750469 | 26.19 |
| A090 Other and unspecified gastroenteritis and colitis of infectious origin | 5091 | 1.42 | 15139 | 2.80 | 12946 | 2.01 | - | 0.00 | 22535 | 4.25 | 10728 | 1.82 | 66439 | 2.32 |
| A099 Gastroenteritis and colitis of unspecified origin | 1834 | 0.51 | 6810 | 1.26 | 5784 | 0.90 |  | 0.00 | 7063 | 1.33 | 1787 | 0.30 | 23278 | 0.81 |
| A90 Dengue fever [classical dengue] | 346 | 0.10 | 1174 | 0.22 | 25408 | 3.94 | 806 | 0.40 | 3481 | 0.66 | 23285 | 3.96 | 54500 | 1.90 |
| A91 Dengue haemorrhagic fever | 68 | 0.02 | 121 | 0.02 | 1348 | 0.21 | 44 | 0.02 | 318 | 0.06 | 724 | 0.12 | 2623 | 0.09 |
| A92 Other mosquito-borne viral fevers | 1133 | 0.32 | 1012 | 0.19 | 1334 | 0.21 | 201 | 0.10 | 226 | 0.04 | 376 | 0.06 | 4282 | 0.15 |
| A920 Chikungunya virus disease | 107 | 0.03 | 28 | 0.01 | 22 | 0.00 | 5 | 0.00 | 15 | 0.00 | 5 | 0.00 | 182 | 0.01 |
| A921 Onyong-nyong fever | 4 | 0.00 | 3 | 0.00 | 6 | 0.00 | 1 | 0.00 | 5 | 0.00 | 4 | 0.00 | 23 | 0.00 |
| A922 Venezuelan equine fever | 4 | 0.00 | 8 | 0.00 | 9 | 0.00 | 3 | 0.00 | 2 | 0.00 |  | 0.00 | 26 | 0.00 |
| A923 West Nile fever | 2 | 0.00 | 2 | 0.00 | 7 | 0.00 | 3 | 0.00 | 11 | 0.00 | 13 | 0.00 | 38 | 0.00 |
| A924 Rift Valley fever | 6 | 0.00 | 8 | 0.00 | 6 | 0.00 | 4 | 0.00 | 27 | 0.01 | 21 | 0.00 | 72 | 0.00 |
| A928 Other specified mosquito-borne viral fevers | 143 | 0.04 | 153 | 0.03 | 91 | 0.01 | 16 | 0.01 | 26 | 0.00 | 90 | 0.02 | 519 | 0.02 |
| A929 Mosquito-borne viral fever, unspecified | 632 | 0.18 | 554 | 0.10 | 968 | 0.15 | 126 | 0.06 | 88 | 0.02 | 298 | 0.05 | 2666 | 0.09 |
| A94 Unspecified arthropod-borne viral fever | 1007 | 0.28 | 1086 | 0.20 | 1342 | 0.21 | 574 | 0.28 | 567 | 0.11 | 634 | 0.11 | 5210 | 0.18 |
| A95 Yellow fever | 6 | 0.00 | 22 | 0.00 | 13 | 0.00 | 2 | 0.00 | 66 | 0.01 | 75 | 0.01 | 184 | 0.01 |
| A950 Sylvatic yellow fever | 3 | 0.00 | 1 | 0.00 |  | 0.00 | 1 | 0.00 | 6 | 0.00 | 4 | 0.00 | 15 | 0.00 |
| A951 Urban yellow fever |  | 0.00 | 5 | 0.00 | 2 | 0.00 |  | 0.00 | 7 | 0.00 | 3 | 0.00 | 17 | 0.00 |
| A959 Yellow fever, unspecified | 3 | 0.00 | 12 | 0.00 | 12 | 0.00 | 6 | 0.00 | 6 | 0.00 | 21 | 0.00 | 60 | 0.00 |
| B15 Acute hepatitis A | 693 | 0.19 | 1307 | 0.24 | 946 | 0.15 | 475 | 0.24 | 509 | 0.10 | 1234 | 0.21 | 5164 | 0.18 |
| B150 Hepatitis A with hepatic coma | 95 | 0.03 | 62 | 0.01 | 59 | 0.01 | 42 | 0.02 | 52 | 0.01 | 124 | 0.02 | 434 | 0.02 |
| B159 Hepatitis A without hepatic coma | 23 | 0.01 | 32 | 0.01 | 30 | 0.00 | 18 | 0.01 | 21 | 0.00 | 35 | 0.01 | 159 | 0.01 |
| B16 Acute hepatitis B | 369 | 0.10 | 706 | 0.13 | 536 | 0.08 | 329 | 0.16 | 350 | 0.07 | 769 | 0.13 | 3059 | 0.11 |
| B160 Acute hepatitis B with delta-agent (coinfection) with hepatic coma | 20 | 0.01 | 30 | 0.01 | 61 | 0.01 | 16 | 0.01 | 21 | 0.00 | 38 | 0.01 | 186 | 0.01 |
| B161 Acute hepatitis B with delta-agent (coinfection) without hepatic coma | 17 | 0.00 | 10 | 0.00 | 11 | 0.00 | 7 | 0.00 | 7 | 0.00 | 19 | 0.00 | 71 | 0.00 |
| B162 Acute hepatitis B without delta-agent with hepatic coma | 6 | 0.00 | 9 | 0.00 | 9 | 0.00 | 1 | 0.00 | 11 | 0.00 | 8 | 0.00 | 44 | 0.00 |
| B169 Acute hepatitis B without delta-agent and without hepatic coma | 42 | 0.01 | 21 | 0.00 | 20 | 0.00 | 14 | 0.01 | 6 | 0.00 | 20 | 0.00 | 123 | 0.00 |
| B17 Other acute viral hepatitis | 624 | 0.17 | 1080 | 0.20 | 808 | 0.13 | 401 | 0.20 | 289 | 0.05 | 647 | 0.11 | 3849 | 0.13 |
| B170 Acute delta-(super)infection of hepatitis B carrier | 4 | 0.00 | 10 | 0.00 | 17 | 0.00 | 6 | 0.00 | 10 | 0.00 | 7 | 0.00 | 54 | 0.00 |
| B171 Acute hepatitis C | 31 | 0.01 | 48 | 0.01 | 63 | 0.01 | 22 | 0.01 | 19 | 0.00 | 62 | 0.01 | 245 | 0.01 |
| B172 Acute hepatitis E | 29 | 0.01 | 69 | 0.01 | 48 | 0.01 | 16 | 0.01 | 6 | 0.00 | 17 | 0.00 | 185 | 0.01 |
| B178 Other specified acute viral hepatitis | 52 | 0.01 | 54 | 0.01 | 50 | 0.01 | 30 | 0.01 | 17 | 0.00 | 45 | 0.01 | 248 | 0.01 |
| B179 Acute viral hepatitis, unspecified | 353 | 0.10 | 648 | 0.12 | 707 | 0.11 | 318 | 0.16 | 215 | 0.04 | 522 | 0.09 | 2763 | 0.10 |
| B18 Chronic viral hepatitis | 100 | 0.03 | 133 | 0.02 | 228 | 0.04 | 145 | 0.07 | 66 | 0.01 | 131 | 0.02 | 803 | 0.03 |
| B180 Chronic viral hepatitis B with delta-agent | 15 | 0.00 | 14 | 0.00 | 20 | 0.00 | 4 | 0.00 | 22 | 0.00 | 82 | 0.01 | 157 | 0.01 |
| B181 Chronic viral hepatitis B without delta-agent | 19 | 0.01 | 10 | 0.00 | 15 | 0.00 | 13 | 0.01 | 30 | 0.01 | 44 | 0.01 | 131 | 0.00 |
| B182 Chronic viral hepatitis C | 14 | 0.00 | 22 | 0.00 | 32 | 0.00 | 19 | 0.01 | 26 | 0.00 | 41 | 0.01 | 154 | 0.01 |
| B188 Other chronic viral hepatitis | 26 | 0.01 | 24 | 0.00 | 17 | 0.00 | 17 | 0.01 | 7 | 0.00 | 25 | 0.00 | 116 | 0.00 |
| B189 Chronic viral hepatitis, unspecified | 31 | 0.01 | 36 | 0.01 | 42 | 0.01 | 11 | 0.01 | 17 | 0.00 | 27 | 0.00 | 164 | 0.01 |
| B19 Unspecified viral hepatitis | 232 | 0.06 | 347 | 0.06 | 270 | 0.04 | 139 | 0.07 | 105 | 0.02 | 178 | 0.03 | 1271 | 0.04 |
| B190 Unspecified viral hepatitis hepatic with coma | 16 | 0.00 | 15 | 0.00 | 17 | 0.00 | 10 | 0.00 | 6 | 0.00 | 23 | 0.00 | 87 | 0.00 |
| B199 Unspecified viral hepatitis without hepatic coma | 100 | 0.03 | 159 | 0.03 | 105 | 0.02 | 40 | 0.02 | 18 | 0.00 | 26 | 0.00 | 448 | 0.02 |
| B50 Plasmodium falciparum malaria | 299 | 0.08 | 274 | 0.05 | 555 | 0.09 | 87 | 0.04 | 135 | 0.03 | 518 | 0.09 | 1868 | 0.07 |
| B500 Plasmodium falciparum malaria with cerebral complications | 19 | 0.01 | 21 | 0.00 | 19 | 0.00 | 3 | 0.00 | 10 | 0.00 | 30 | 0.01 | 102 | 0.00 |
| B508 Other severe and complicated Plasmodium falciparum malaria | 94 | 0.03 | 79 | 0.01 | 98 | 0.02 | 51 | 0.03 | 38 | 0.01 | 89 | 0.02 | 449 | 0.02 |
| B509 Plasmodium falciparum malaria, unspecified | 79 | 0.02 | 126 | 0.02 | 433 | 0.07 | 17 | 0.01 | 36 | 0.01 | 145 | 0.02 | 836 | 0.03 |
| B51 Plasmodium vivax malaria | 75 | 0.02 | 48 | 0.01 | 103 | 0.02 | 13 | 0.01 | 49 | 0.01 | 321 | 0.05 | 609 | 0.02 |
| B510 Plasmodium vivax malaria with rupture of spleen | 3 | 0.00 | 6 | 0.00 | 3 | 0.00 | 3 | 0.00 | 5 | 0.00 | 13 | 0.00 | 33 | 0.00 |
| B518 Plasmodium vivax malaria with other complications | 3 | 0.00 | 2 | 0.00 | 6 | 0.00 |  | 0.00 | 1 | 0.00 | 3 | 0.00 | 15 | 0.00 |
| B519 Plasmodium vivax malaria without complication | 4 | 0.00 | 7 | 0.00 | 6 | 0.00 |  | 0.00 | 1 | 0.00 | 14 | 0.00 | 32 | 0.00 |
| B54 Unspecified malaria | 59 | 0.02 | 33 | 0.01 | 42 | 0.01 | 12 | 0.01 | 21 | 0.00 | 77 | 0.01 | 244 | 0.01 |
| B55 Leishmaniasis | 70 | 0.02 | 91 | 0.02 | 81 | 0.01 | 24 | 0.01 | 9 | 0.00 | 5 | 0.00 | 280 | 0.01 |
| B550 Visceral leishmaniasis | 83 | 0.02 | 68 | 0.01 | 81 | 0.01 | 78 | 0.04 | 26 | 0.00 | 19 | 0.00 | 355 | 0.01 |
| B551 Cutaneous leishmaniasis | 1 | 0.00 | 1 | 0.00 | 2 | 0.00 | 4 | 0.00 | 1 | 0.00 | 2 | 0.00 | 11 | 0.00 |
| B552 Mucocutaneous leishmaniasis | 2 | 0.00 |  | 0.00 | 1 | 0.00 | 1 | 0.00 | 1 | 0.00 | 4 | 0.00 | 9 | 0.00 |
| B559 Leishmaniasis, unspecified | 6 | 0.00 | 4 | 0.00 | 7 | 0.00 | 2 | 0.00 | 7 | 0.00 | 3 | 0.00 | 29 | 0.00 |
| B86 Scabies | 71 | 0.02 | 144 | 0.03 | 147 | 0.02 | 84 | 0.04 | 129 | 0.02 | 208 | 0.04 | 783 | 0.03 |
| D02 Carcinoma in situ of middle ear and respiratory system | 68 | 0.02 | 178 | 0.03 | 111 | 0.02 | 109 | 0.05 | 199 | 0.04 | 312 | 0.05 | 977 | 0.03 |
| D020 Larynx | 11 | 0.00 | 25 | 0.00 | 3 | 0.00 | 3 | 0.00 | 10 | 0.00 | 27 | 0.00 | 79 | 0.00 |
| D021 Trachea | 7 | 0.00 | 11 | 0.00 | 7 | 0.00 | 7 | 0.00 | 3 | 0.00 | 13 | 0.00 | 48 | 0.00 |
| D022 Bronchus and lung | 149 | 0.04 | 68 | 0.01 | 39 | 0.01 | 26 | 0.01 | 48 | 0.01 | 65 | 0.01 | 395 | 0.01 |
| D023 Other parts of respiratory system | 73 | 0.02 | 72 | 0.01 | 100 | 0.02 | 136 | 0.07 | 42 | 0.01 | 127 | 0.02 | 550 | 0.02 |
| D024 Respiratory system, unspecified | 322 | 0.09 | 330 | 0.06 | 430 | 0.07 | 236 | 0.12 | 331 | 0.06 | 549 | 0.09 | 2198 | 0.08 |
| D04 Carcinoma in situ of skin | 21 | 0.01 | 38 | 0.01 | 18 | 0.00 | 19 | 0.01 | 39 | 0.01 | 81 | 0.01 | 216 | 0.01 |
| D040 Skin of lip |  | 0.00 |  | 0.00 |  | 0.00 | 1 | 0.00 |  | 0.00 | 2 | 0.00 | 3 | 0.00 |
| D041 Skin of eyelid, including canthus | 6 | 0.00 | 50 | 0.01 | 32 | 0.00 | 3 | 0.00 | 19 | 0.00 | 20 | 0.00 | 130 | 0.00 |
| D042 Skin of ear and external auricular canal | 21 | 0.01 | 19 | 0.00 | 6 | 0.00 | 3 | 0.00 | 11 | 0.00 | 20 | 0.00 | 80 | 0.00 |
| D043 Skin of other and unspecified parts of face | 12 | 0.00 | 4 | 0.00 | 7 | 0.00 | 4 | 0.00 | 2 | 0.00 | 8 | 0.00 | 37 | 0.00 |
| D044 Skin of scalp and neck | 51 | 0.01 | 47 | 0.01 | 41 | 0.01 | 36 | 0.02 | 27 | 0.01 | 51 | 0.01 | 253 | 0.01 |
| D045 Skin of trunk |  | 0.00 | 2 | 0.00 | 2 | 0.00 | 2 | 0.00 | 2 | 0.00 | 3 | 0.00 | 11 | 0.00 |
| D046 Skin of upper limb, including shoulder | 8 | 0.00 | 17 | 0.00 | 17 | 0.00 | 16 | 0.01 | 28 | 0.01 | 23 | 0.00 | 109 | 0.00 |
| D047 Skin of lower limb, including hip | 17 | 0.00 | 30 | 0.01 | 72 | 0.01 | 54 | 0.03 | 5 | 0.00 | 19 | 0.00 | 197 | 0.01 |
| D048 Skin of other sites |  | 0.00 | 4 | 0.00 | 33 | 0.01 | 14 | 0.01 | 7 | 0.00 | 7 | 0.00 | 65 | 0.00 |
| D049 Skin, unspecified | 1 | 0.00 | 16 | 0.00 | 21 | 0.00 | 21 | 0.01 | 11 | 0.00 | 35 | 0.01 | 105 | 0.00 |
| E10 Insulin-dependent diabetes mellitus | 7159 | 1.99 | 10487 | 1.94 | 12812 | 1.99 | 9713 | 4.82 | 13228 | 2.49 | 20369 | 3.46 | 73768 | 2.57 |
| E100 Insulin-dependent diabetes mellitus with coma | 355 | 0.10 | 261 | 0.05 | 263 | 0.04 | 233 | 0.12 | 265 | 0.05 | 490 | 0.08 | 1867 | 0.07 |
| E101 Insulin-dependent diabetes mellitus with ketoacidosis | 115 | 0.03 | 171 | 0.03 | 211 | 0.03 | 142 | 0.07 | 144 | 0.03 | 292 | 0.05 | 1075 | 0.04 |
| E102 Insulin-dependent diabetes mellitus with renal complications | 176 | 0.05 | 153 | 0.03 | 178 | 0.03 | 99 | 0.05 | 59 | 0.01 | 205 | 0.03 | 870 | 0.03 |
| E103 Insulin-dependent diabetes mellitus with ophthalmic complications | 49 | 0.01 | 47 | 0.01 | 58 | 0.01 | 63 | 0.03 | 30 | 0.01 | 68 | 0.01 | 315 | 0.01 |
| E104 Insulin-dependent diabetes mellitus with neurological complications | 60 | 0.02 | 59 | 0.01 | 55 | 0.01 | 41 | 0.02 | 63 | 0.01 | 76 | 0.01 | 354 | 0.01 |
| E105 Insulin-dependent diabetes mellitus with peripheral circulatory compli | 40 | 0.01 | 49 | 0.01 | 55 | 0.01 | 103 | 0.05 | 214 | 0.04 | 89 | 0.02 | 550 | 0.02 |
| E106 Insulin-dependent diabetes mellitus with other specified complications | 141 | 0.04 | 198 | 0.04 | 535 | 0.08 | 307 | 0.15 | 197 | 0.04 | 403 | 0.07 | 1781 | 0.06 |
| E107 Insulin-dependent diabetes mellitus with multiple complications | 139 | 0.04 | 330 | 0.06 | 317 | 0.05 | 185 | 0.09 | 189 | 0.04 | 403 | 0.07 | 1563 | 0.05 |
| E108 Insulin-dependent diabetes mellitus with unspecified complications | 139 | 0.04 | 259 | 0.05 | 336 | 0.05 | 75 | 0.04 | 111 | 0.02 | 106 | 0.02 | 1026 | 0.04 |
| E109 Insulin-dependent diabetes mellitus without complications | 133 | 0.04 | 224 | 0.04 | 129 | 0.02 | 42 | 0.02 | 51 | 0.01 | 52 | 0.01 | 631 | 0.02 |
| E11 Non-insulin-dependent diabetes mellitus | 1570 | 0.44 | 2606 | 0.48 | 3895 | 0.60 | 3268 | 1.62 | 4079 | 0.77 | 6751 | 1.15 | 22169 | 0.77 |
| E110 Non-insulin-dependent diabetes mellitus with coma | 103 | 0.03 | 272 | 0.05 | 315 | 0.05 | 475 | 0.24 | 178 | 0.03 | 131 | 0.02 | 1474 | 0.05 |
| E111 Non-insulin-dependent diabetes mellitus with ketoacidosis | 48 | 0.01 | 74 | 0.01 | 66 | 0.01 | 74 | 0.04 | 120 | 0.02 | 404 | 0.07 | 786 | 0.03 |
| E112 Non-insulin-dependent diabetes mellitus with renal complications | 36 | 0.01 | 28 | 0.01 | 48 | 0.01 | 47 | 0.02 | 33 | 0.01 | 158 | 0.03 | 350 | 0.01 |
| E113 Non-insulin-dependent diabetes mellitus with ophthalmic complications | 10 | 0.00 | 7 | 0.00 | 26 | 0.00 | 33 | 0.02 | 15 | 0.00 | 16 | 0.00 | 107 | 0.00 |
| E114 Non-insulin-dependent diabetes mellitus with neurological complication | 14 | 0.00 | 19 | 0.00 | 28 | 0.00 | 22 | 0.01 | 51 | 0.01 | 173 | 0.03 | 307 | 0.01 |
| E115 Non-insulin-dependent diabetes mellitus with peripheral circulatory co | 176 | 0.05 | 187 | 0.03 | 115 | 0.02 | 101 | 0.05 | 77 | 0.01 | 97 | 0.02 | 753 | 0.03 |
| E116 Non-insulin-dependent diabetes mellitus with other specified complicat | 159 | 0.04 | 248 | 0.05 | 323 | 0.05 | 245 | 0.12 | 417 | 0.08 | 336 | 0.06 | 1728 | 0.06 |
| E117 Non-insulin-dependent diabetes mellitus with multiple complications | 62 | 0.02 | 96 | 0.02 | 74 | 0.01 | 45 | 0.02 | 51 | 0.01 | 97 | 0.02 | 425 | 0.01 |
| E118 Non-insulin-dependent diabetes mellitus with unspecified complications | 38 | 0.01 | 29 | 0.01 | 37 | 0.01 | 33 | 0.02 | 28 | 0.01 | 36 | 0.01 | 201 | 0.01 |
| E119 Non-insulin-dependent diabetes mellitus without complications | 74 | 0.02 | 35 | 0.01 | 138 | 0.02 | 198 | 0.10 | 246 | 0.05 | 767 | 0.13 | 1458 | 0.05 |
| E12 Malnutrition-related diabetes mellitus | 88 | 0.02 | 263 | 0.05 | 235 | 0.04 | 228 | 0.11 | 417 | 0.08 | 397 | 0.07 | 1628 | 0.06 |
| E120 Malnutrition-related diabetes mellitus with coma | 15 | 0.00 | 30 | 0.01 | 52 | 0.01 | 16 | 0.01 | 32 | 0.01 | 17 | 0.00 | 162 | 0.01 |
| E121 Malnutrition-related diabetes mellitus with ketoacidosis | 17 | 0.00 | 14 | 0.00 | 32 | 0.00 | 17 | 0.01 | 15 | 0.00 | 19 | 0.00 | 114 | 0.00 |
| E122 Malnutrition-related diabetes mellitus with renal complications | 4 | 0.00 | 11 | 0.00 | 11 | 0.00 | 2 | 0.00 | 7 | 0.00 | 16 | 0.00 | 51 | 0.00 |
| E123 Malnutrition-related diabetes mellitus with ophthalmic complications | 5 | 0.00 | 7 | 0.00 | 7 | 0.00 | 2 | 0.00 | 3 | 0.00 | 1 | 0.00 | 25 | 0.00 |
| E124 Malnutrition-related diabetes mellitus with neurological complications | 5 | 0.00 | 17 | 0.00 | 12 | 0.00 | 9 | 0.00 | 4 | 0.00 | 9 | 0.00 | 56 | 0.00 |
| E125 Malnutrition-related diabetes mellitus with peripheral circulatory com | 9 | 0.00 | 6 | 0.00 | 19 | 0.00 | 17 | 0.01 | 6 | 0.00 | 7 | 0.00 | 64 | 0.00 |
| E126 Malnutrition-related diabetes mellitus with other specified complicati | 11 | 0.00 | 8 | 0.00 | 7 | 0.00 | 5 | 0.00 | 3 | 0.00 | 3 | 0.00 | 37 | 0.00 |
| E127 Malnutrition-related diabetes mellitus with multiple complications |  | 0.00 | 7 | 0.00 | 3 | 0.00 | 3 | 0.00 | 5 | 0.00 | 5 | 0.00 | 23 | 0.00 |
| E128 Malnutrition-related diabetes mellitus with unspecified complications |  | 0.00 | 5 | 0.00 | 8 | 0.00 | 14 | 0.01 | 6 | 0.00 | 5 | 0.00 | 38 | 0.00 |
| E129 Malnutrition-related diabetes mellitus without complications | 4 | 0.00 | 1 | 0.00 | 6 | 0.00 | 6 | 0.00 | 2 | 0.00 | 2 | 0.00 | 21 | 0.00 |
| E13 Other specified diabetes mellitus | 77 | 0.02 | 300 | 0.06 | 300 | 0.05 | 245 | 0.12 | 362 | 0.07 | 934 | 0.16 | 2218 | 0.08 |
| E130 Other specified diabetes mellitus with coma | 12 | 0.00 | 6 | 0.00 | 8 | 0.00 | 6 | 0.00 | 13 | 0.00 | 23 | 0.00 | 68 | 0.00 |
| E131 Other specified diabetes mellitus with ketoacidosis | 13 | 0.00 | 14 | 0.00 | 13 | 0.00 | 20 | 0.01 | 20 | 0.00 | 41 | 0.01 | 121 | 0.00 |
| E132 Other specified diabetes mellitus with renal complications | 17 | 0.00 | 20 | 0.00 | 21 | 0.00 | 9 | 0.00 | 20 | 0.00 | 15 | 0.00 | 102 | 0.00 |
| E133 Other specified diabetes mellitus with ophthalmic complications | 2 | 0.00 | 4 | 0.00 | 3 | 0.00 | 2 | 0.00 | 8 | 0.00 | 7 | 0.00 | 26 | 0.00 |
| E134 Other specified diabetes mellitus with neurological complications | 5 | 0.00 | 6 | 0.00 | 3 | 0.00 | 4 | 0.00 | 14 | 0.00 | 21 | 0.00 | 53 | 0.00 |
| E135 Other specified diabetes mellitus with peripheral circulatory complica | 5 | 0.00 | 8 | 0.00 | 11 | 0.00 | 17 | 0.01 | 81 | 0.02 | 23 | 0.00 | 145 | 0.01 |
| E136 Other specified diabetes mellitus with other specified complications | 1 | 0.00 | 14 | 0.00 | 65 | 0.01 | 10 | 0.00 | 7 | 0.00 | 16 | 0.00 | 113 | 0.00 |
| E137 Other specified diabetes mellitus with multiple complications | 8 | 0.00 | 29 | 0.01 | 18 | 0.00 | 14 | 0.01 | 10 | 0.00 | 10 | 0.00 | 89 | 0.00 |
| E138 Other specified diabetes mellitus with unspecified complications | 34 | 0.01 | 76 | 0.01 | 70 | 0.01 | 77 | 0.04 | 209 | 0.04 | 587 | 0.10 | 1053 | 0.04 |
| E139 Other specified diabetes mellitus without complications | 2 | 0.00 | 15 | 0.00 | 7 | 0.00 | 11 | 0.01 | 17 | 0.00 | 42 | 0.01 | 94 | 0.00 |
| E14 Unspecified diabetes mellitus | 3302 | 0.92 | 4490 | 0.83 | 6259 | 0.97 | 4984 | 2.47 | 5407 | 1.02 | 7899 | 1.34 | 32341 | 1.13 |
| E140 Unspecified diabetes mellitus with coma | 123 | 0.03 | 61 | 0.01 | 92 | 0.01 | 145 | 0.07 | 89 | 0.02 | 65 | 0.01 | 575 | 0.02 |
| E141 Unspecified diabetes mellitus with ketoacidosis | 119 | 0.03 | 23 | 0.00 | 62 | 0.01 | 115 | 0.06 | 160 | 0.03 | 273 | 0.05 | 752 | 0.03 |
| E142 Unspecified diabetes mellitus with renal complications | 24 | 0.01 | 16 | 0.00 | 37 | 0.01 | 29 | 0.01 | 22 | 0.00 | 28 | 0.00 | 156 | 0.01 |
| E143 Unspecified diabetes mellitus with ophthalmic complications | 5 | 0.00 | 10 | 0.00 | 15 | 0.00 | 31 | 0.02 | 7 | 0.00 | 3 | 0.00 | 71 | 0.00 |
| E144 Unspecified diabetes mellitus with neurological complications | 4 | 0.00 | 17 | 0.00 | 9 | 0.00 | 9 | 0.00 | 9 | 0.00 | 18 | 0.00 | 66 | 0.00 |
| E145 Unspecified diabetes mellitus with peripheral circulatory complication | 67 | 0.02 | 113 | 0.02 | 98 | 0.02 | 122 | 0.06 | 129 | 0.02 | 73 | 0.01 | 602 | 0.02 |
| E146 Unspecified diabetes mellitus with other specified complications | 94 | 0.03 | 148 | 0.03 | 128 | 0.02 | 96 | 0.05 | 30 | 0.01 | 93 | 0.02 | 589 | 0.02 |
| E147 Unspecified diabetes mellitus with multiple complications | 30 | 0.01 | 82 | 0.02 | 63 | 0.01 | 79 | 0.04 | 29 | 0.01 | 62 | 0.01 | 345 | 0.01 |
| E148 Unspecified diabetes mellitus with unspecified complications | 18 | 0.01 | 29 | 0.01 | 28 | 0.00 | 23 | 0.01 | 22 | 0.00 | 40 | 0.01 | 160 | 0.01 |
| E149 Unspecified diabetes mellitus without complications | 32 | 0.01 | 57 | 0.01 | 76 | 0.01 | 96 | 0.05 | 257 | 0.05 | 326 | 0.06 | 844 | 0.03 |
| E40 Kwashiorkor | 53 | 0.01 | 56 | 0.01 | 64 | 0.01 | 112 | 0.06 | 153 | 0.03 | 119 | 0.02 | 557 | 0.02 |
| E41 Nutritional marasmus | 60 | 0.02 | 63 | 0.01 | 71 | 0.01 | 50 | 0.02 | 174 | 0.03 | 251 | 0.04 | 669 | 0.02 |
| E42 Marasmic kwashiorkor | 29 | 0.01 | 8 | 0.00 | 7 | 0.00 | 31 | 0.02 | 113 | 0.02 | 104 | 0.02 | 292 | 0.01 |
| E43 Unspecified severe protein-energy malnutrition | 1359 | 0.38 | 1658 | 0.31 | 1914 | 0.30 | 1733 | 0.86 | 2414 | 0.45 | 2410 | 0.41 | 11488 | 0.40 |
| E44 Protein-energy malnutrition of moderate and mild degree | 182 | 0.05 | 230 | 0.04 | 368 | 0.06 | 383 | 0.19 | 727 | 0.14 | 498 | 0.08 | 2388 | 0.08 |
| E440 Moderate protein-energy malnutrition | 144 | 0.04 | 134 | 0.02 | 181 | 0.03 | 199 | 0.10 | 316 | 0.06 | 281 | 0.05 | 1255 | 0.04 |
| E441 Mild protein-energy malnutrition | 182 | 0.05 | 415 | 0.08 | 265 | 0.04 | 276 | 0.14 | 396 | 0.07 | 465 | 0.08 | 1999 | 0.07 |
| E45 Retarded development following protein-energy malnutrition | 25 | 0.01 | 41 | 0.01 | 68 | 0.01 | 33 | 0.02 | 24 | 0.00 | 31 | 0.01 | 222 | 0.01 |
| E46 Unspecified protein-energy malnutrition | 458 | 0.13 | 1416 | 0.26 | 1864 | 0.29 | 1404 | 0.70 | 1761 | 0.33 | 2089 | 0.35 | 8992 | 0.31 |
| F32 Depressive episode | 131 | 0.04 | 91 | 0.02 | 57 | 0.01 | 35 | 0.02 | 73 | 0.01 | 183 | 0.03 | 570 | 0.02 |
| F320 Mild depressive episode | 49 | 0.01 | 119 | 0.02 | 8 | 0.00 | 6 | 0.00 | 3 | 0.00 | 10 | 0.00 | 195 | 0.01 |
| F321 Moderate depressive episode | 4 | 0.00 | 3 | 0.00 | 6 | 0.00 | 4 | 0.00 | 5 | 0.00 | 7 | 0.00 | 29 | 0.00 |
| F322 Severe depressive episode without psychotic symptoms | 9 | 0.00 | 7 | 0.00 | 7 | 0.00 | 10 | 0.00 | 8 | 0.00 | 18 | 0.00 | 59 | 0.00 |
| F323 Severe depressive episode with psychotic symptoms | 10 | 0.00 | 9 | 0.00 | 11 | 0.00 | 2 | 0.00 | 7 | 0.00 | 25 | 0.00 | 64 | 0.00 |
| F328 Other depressive episodes | 45 | 0.01 | 12 | 0.00 | 3 | 0.00 | 7 | 0.00 | 12 | 0.00 | 12 | 0.00 | 91 | 0.00 |
| F329 Depressive episode, unspecified | 47 | 0.01 | 27 | 0.00 | 24 | 0.00 | 20 | 0.01 | 44 | 0.01 | 35 | 0.01 | 197 | 0.01 |
| F33 Recurrent depressive disorder | 39 | 0.01 | 56 | 0.01 | 65 | 0.01 | 36 | 0.02 | 32 | 0.01 | 162 | 0.03 | 390 | 0.01 |
| F330 Recurrent depressive disorder, current episode mild | 4 | 0.00 | 3 | 0.00 | 11 | 0.00 | 5 | 0.00 | 1 | 0.00 | 5 | 0.00 | 29 | 0.00 |
| F331 Recurrent depressive disorder, current episode moderate | 5 | 0.00 | 1 | 0.00 | 10 | 0.00 | 8 | 0.00 | 5 | 0.00 | 13 | 0.00 | 42 | 0.00 |
| F332 Recurrent depressive disorder, current episode severe without psychoti |  | 0.00 | 2 | 0.00 | 6 | 0.00 | 2 | 0.00 |  | 0.00 | 3 | 0.00 | 13 | 0.00 |
| F333 Recurrent depressive disorder, current episode severe with psychotic s | 1 | 0.00 | 4 | 0.00 | 6 | 0.00 | 7 | 0.00 | 2 | 0.00 | 4 | 0.00 | 24 | 0.00 |
| F334 Recurrent depressive disorder, currently in remission | 2 | 0.00 |  | 0.00 | 2 | 0.00 | 2 | 0.00 | 1 | 0.00 | 3 | 0.00 | 10 | 0.00 |
| F338 Other recurrent depressive disorders | 12 | 0.00 | 6 | 0.00 | 8 | 0.00 | 5 | 0.00 | 4 | 0.00 | 8 | 0.00 | 43 | 0.00 |
| F339 Recurrent depressive disorder, unspecified | 9 | 0.00 | 12 | 0.00 | 6 | 0.00 | 10 | 0.00 | 22 | 0.00 | 19 | 0.00 | 78 | 0.00 |
| F34 Persistent mood [affective] disorders | 2 | 0.00 | 3 | 0.00 | 13 | 0.00 | 11 | 0.01 | 3 | 0.00 | 6 | 0.00 | 38 | 0.00 |
| F340 Cyclothymia | 2 | 0.00 | 2 | 0.00 | 3 | 0.00 | 4 | 0.00 | 2 | 0.00 | 4 | 0.00 | 17 | 0.00 |
| F341 Dysthymia | 28 | 0.01 | 7 | 0.00 | 7 | 0.00 | 4 | 0.00 | 5 | 0.00 | 30 | 0.01 | 81 | 0.00 |
| F348 Other persistent mood [affective] disorders |  | 0.00 | 1 | 0.00 | 1 | 0.00 | 1 | 0.00 | 1 | 0.00 | 6 | 0.00 | 10 | 0.00 |
| F349 Persistent mood [affective] disorder, unspecified | 2 | 0.00 | 2 | 0.00 | 1 | 0.00 | 2 | 0.00 | 1 | 0.00 | 2 | 0.00 | 10 | 0.00 |
| F40 Phobic anxiety disorders | 1695 | 0.47 | 1710 | 0.32 | 1871 | 0.29 | 1356 | 0.67 | 1643 | 0.31 | 1432 | 0.24 | 9707 | 0.34 |
| F400 Agoraphobia | 37 | 0.01 | 11 | 0.00 | 7 | 0.00 | 6 | 0.00 | 7 | 0.00 | 4 | 0.00 | 72 | 0.00 |
| F401 Social phobias | 16 | 0.00 | 10 | 0.00 | 12 | 0.00 | 9 | 0.00 | 6 | 0.00 | 11 | 0.00 | 64 | 0.00 |
| F402 Specific (isolated) phobias | 10 | 0.00 | 6 | 0.00 | 9 | 0.00 | 8 | 0.00 | 10 | 0.00 | 4 | 0.00 | 47 | 0.00 |
| F408 Other phobic anxiety disorders | 177 | 0.05 | 287 | 0.05 | 433 | 0.07 | 243 | 0.12 | 175 | 0.03 | 157 | 0.03 | 1472 | 0.05 |
| F409 Phobic anxiety disorder, unspecified | 151 | 0.04 | 143 | 0.03 | 409 | 0.06 | 233 | 0.12 | 145 | 0.03 | 136 | 0.02 | 1217 | 0.04 |
| F41 Other anxiety disorders | 12871 | 3.59 | 19930 | 3.68 | 21398 | 3.32 | 12305 | 6.10 | 15794 | 2.98 | 20565 | 3.49 | 102863 | 3.59 |
| F410 Panic disorder [episodic paroxysmal anxiety] | 777 | 0.22 | 874 | 0.16 | 1113 | 0.17 | 571 | 0.28 | 812 | 0.15 | 1126 | 0.19 | 5273 | 0.18 |
| F411 Generalized anxiety disorder | 23131 | 6.45 | 33802 | 6.25 | 39901 | 6.19 | 26886 | 13.33 | 33658 | 6.34 | 46248 | 7.86 | 203626 | 7.11 |
| F412 Mixed anxiety and depressive disorder | 661 | 0.18 | 1194 | 0.22 | 1322 | 0.21 | 485 | 0.24 | 534 | 0.10 | 873 | 0.15 | 5069 | 0.18 |
| F413 Other mixed anxiety disorders | 167 | 0.05 | 128 | 0.02 | 142 | 0.02 | 70 | 0.03 | 65 | 0.01 | 90 | 0.02 | 662 | 0.02 |
| F418 Other specified anxiety disorders | 185 | 0.05 | 250 | 0.05 | 158 | 0.02 | 79 | 0.04 | 148 | 0.03 | 624 | 0.11 | 1444 | 0.05 |
| F419 Anxiety disorder, unspecified | 9792 | 2.73 | 13774 | 2.55 | 12979 | 2.01 | 7549 | 3.74 | 6871 | 1.29 | 6912 | 1.17 | 57877 | 2.02 |
| F43 Reaction to severe stress, and adjustment disorders | 77 | 0.02 | 271 | 0.05 | 418 | 0.06 | 323 | 0.16 | 269 | 0.05 | 473 | 0.08 | 1831 | 0.06 |
| F430 Acute stress reaction | 419 | 0.12 | 408 | 0.08 | 299 | 0.05 | 179 | 0.09 | 230 | 0.04 | 375 | 0.06 | 1910 | 0.07 |
| F431 Post-traumatic stress disorder | 379 | 0.11 | 649 | 0.12 | 709 | 0.11 | 574 | 0.28 | 822 | 0.15 | 1305 | 0.22 | 4438 | 0.15 |
| F432 Adjustment disorders | 116 | 0.03 | 263 | 0.05 | 138 | 0.02 | 78 | 0.04 | 79 | 0.01 | 34 | 0.01 | 708 | 0.02 |
| F438 Other reactions to severe stress | 14 | 0.00 | 15 | 0.00 | 18 | 0.00 | 11 | 0.01 | 12 | 0.00 | 22 | 0.00 | 92 | 0.00 |
| F439 Reaction to severe stress, unspecified | 21 | 0.01 | 12 | 0.00 | 17 | 0.00 | 15 | 0.01 | 34 | 0.01 | 33 | 0.01 | 132 | 0.00 |
| F44 Dissociative [conversion] disorders | 10972 | 3.06 | 13346 | 2.47 | 14980 | 2.32 | 9198 | 4.56 | 9807 | 1.85 | 11598 | 1.97 | 69901 | 2.44 |
| F440 Dissociative amnesia | 186 | 0.05 | 195 | 0.04 | 207 | 0.03 | 108 | 0.05 | 60 | 0.01 | 63 | 0.01 | 819 | 0.03 |
| F441 Dissociative fugue | 237 | 0.07 | 389 | 0.07 | 179 | 0.03 | 201 | 0.10 | 185 | 0.03 | 156 | 0.03 | 1347 | 0.05 |
| F442 Dissociative stupor | 23 | 0.01 | 126 | 0.02 | 24 | 0.00 | 9 | 0.00 | 8 | 0.00 | 11 | 0.00 | 201 | 0.01 |
| F443 Trance and possession disorders | 12 | 0.00 | 34 | 0.01 | 25 | 0.00 | 7 | 0.00 | 11 | 0.00 | 6 | 0.00 | 95 | 0.00 |
| F444 Dissociative motor disorders | 357 | 0.10 | 323 | 0.06 | 392 | 0.06 | 296 | 0.15 | 406 | 0.08 | 589 | 0.10 | 2363 | 0.08 |
| F445 Dissociative convulsions | 967 | 0.27 | 1192 | 0.22 | 1084 | 0.17 | 898 | 0.45 | 960 | 0.18 | 1274 | 0.22 | 6375 | 0.22 |
| F446 Dissociative anaesthesia and sensory loss | 21 | 0.01 | 10 | 0.00 | 11 | 0.00 | 6 | 0.00 | 6 | 0.00 | 18 | 0.00 | 72 | 0.00 |
| F447 Mixed dissociative [conversion] disorders | 646 | 0.18 | 782 | 0.14 | 459 | 0.07 | 213 | 0.11 | 428 | 0.08 | 537 | 0.09 | 3065 | 0.11 |
| F448 Other dissociative [conversion] disorders | 257 | 0.07 | 336 | 0.06 | 541 | 0.08 | 349 | 0.17 | 501 | 0.09 | 938 | 0.16 | 2922 | 0.10 |
| F449 Dissociative [conversion] disorder, unspecified | 3273 | 0.91 | 3091 | 0.57 | 3062 | 0.48 | 1802 | 0.89 | 1478 | 0.28 | 2686 | 0.46 | 15392 | 0.54 |
| F45 Somatoform disorders | 3244 | 0.90 | 4315 | 0.80 | 4281 | 0.66 | 2477 | 1.23 | 3097 | 0.58 | 3973 | 0.67 | 21387 | 0.75 |
| F450 Somatization disorder | 151 | 0.04 | 226 | 0.04 | 239 | 0.04 | 96 | 0.05 | 91 | 0.02 | 119 | 0.02 | 922 | 0.03 |
| F451 Undifferentiated somatoform disorder | 27 | 0.01 | 55 | 0.01 | 73 | 0.01 | 49 | 0.02 | 42 | 0.01 | 69 | 0.01 | 315 | 0.01 |
| F452 Hypochondriacal disorder | 68 | 0.02 | 132 | 0.02 | 77 | 0.01 | 69 | 0.03 | 86 | 0.02 | 120 | 0.02 | 552 | 0.02 |
| F453 Somatoform autonomic dysfunction | 27 | 0.01 | 4 | 0.00 | 8 | 0.00 | 7 | 0.00 | 2 | 0.00 | 34 | 0.01 | 82 | 0.00 |
| F454 Persistent somatoform pain disorder | 339 | 0.09 | 502 | 0.09 | 505 | 0.08 | 277 | 0.14 | 305 | 0.06 | 684 | 0.12 | 2612 | 0.09 |
| F458 Other somatoform disorders | 23 | 0.01 | 46 | 0.01 | 49 | 0.01 | 34 | 0.02 | 30 | 0.01 | 38 | 0.01 | 220 | 0.01 |
| F459 Somatoform disorder, unspecified | 467 | 0.13 | 475 | 0.09 | 428 | 0.07 | 761 | 0.38 | 689 | 0.13 | 549 | 0.09 | 3369 | 0.12 |
| F70 Mild mental retardation | 22 | 0.01 | 114 | 0.02 | 147 | 0.02 | 107 | 0.05 | 123 | 0.02 | 81 | 0.01 | 594 | 0.02 |
| F700 Mild mental retardation with the statement of no, or minimal, impairme | 3 | 0.00 | 4 | 0.00 | 3 | 0.00 | 2 | 0.00 | 5 | 0.00 | 2 | 0.00 | 19 | 0.00 |
| F701 Mild mental retardation, significant impairment of behaviour requiring | 2 | 0.00 | 6 | 0.00 | 5 | 0.00 | 1 | 0.00 | 13 | 0.00 | 12 | 0.00 | 39 | 0.00 |
| F708 Mild mental retardation, other impairments of behaviour | 3 | 0.00 | 4 | 0.00 | 14 | 0.00 | 18 | 0.01 | 5 | 0.00 | 2 | 0.00 | 46 | 0.00 |
| F709 Mild mental retardation without mention of impairment of behaviour | 2 | 0.00 | 1 | 0.00 | 1 | 0.00 | 2 | 0.00 | 2 | 0.00 | 3 | 0.00 | 11 | 0.00 |
| F80 Specific developmental disorders of speech and language | 5 | 0.00 | 6 | 0.00 | 6 | 0.00 | 6 | 0.00 | 13 | 0.00 | 20 | 0.00 | 56 | 0.00 |
| F800 Specific speech articulation disorder | 2 | 0.00 | 34 | 0.01 | 7 | 0.00 | 5 | 0.00 | 5 | 0.00 | 6 | 0.00 | 59 | 0.00 |
| F801 Expressive language disorder | 1 | 0.00 | 2 | 0.00 |  | 0.00 | 1 | 0.00 | 4 | 0.00 | 12 | 0.00 | 20 | 0.00 |
| F802 Receptive language disorder | 2 | 0.00 | 2 | 0.00 |  | 0.00 | 1 | 0.00 |  | 0.00 | 3 | 0.00 | 8 | 0.00 |
| F803 Acquired aphasia with epilepsy [Landau-Kleffner] | 28 | 0.01 | 40 | 0.01 | 30 | 0.00 | 35 | 0.02 | 30 | 0.01 | 35 | 0.01 | 198 | 0.01 |
| F808 Other developmental disorders of speech and language | 6 | 0.00 | 5 | 0.00 | 3 | 0.00 | 6 | 0.00 | 15 | 0.00 | 9 | 0.00 | 44 | 0.00 |
| F809 Developmental disorder of speech and language, unspecified | 3 | 0.00 | 5 | 0.00 | 4 | 0.00 | 7 | 0.00 | 5 | 0.00 | 5 | 0.00 | 29 | 0.00 |
| F81 Specific developmental disorders of scholastic skills | 1 | 0.00 | 2 | 0.00 | 1 | 0.00 | 2 | 0.00 | 3 | 0.00 | 4 | 0.00 | 13 | 0.00 |
| F810 Specific reading disorder |  | 0.00 | 1 | 0.00 | 1 | 0.00 |  | 0.00 | 1 | 0.00 | 3 | 0.00 | 6 | 0.00 |
| F811 Specific spelling disorder |  | 0.00 | 3 | 0.00 |  | 0.00 | 1 | 0.00 | 1 | 0.00 | 4 | 0.00 | 9 | 0.00 |
| F812 Specific disorder of arithmetical skills |  | 0.00 | 8 | 0.00 | 3 | 0.00 |  | 0.00 | 3 | 0.00 | 3 | 0.00 | 17 | 0.00 |
| F813 Mixed disorder of scholastic skills |  | 0.00 |  | 0.00 | 5 | 0.00 | 1 | 0.00 |  | 0.00 |  | 0.00 | 6 | 0.00 |
| F818 Other developmental disorders of scholastic skills | 1 | 0.00 | 1 | 0.00 |  | 0.00 |  | 0.00 |  | 0.00 | 3 | 0.00 | 5 | 0.00 |
| F819 Developmental disorder of scholastic skills, unspecified | 1 | 0.00 | 1 | 0.00 | 3 | 0.00 |  | 0.00 | 1 | 0.00 | 3 | 0.00 | 9 | 0.00 |
| F82 Specific developmental disorder of motor function | 3 | 0.00 | 3 | 0.00 | 13 | 0.00 | 24 | 0.01 | 17 | 0.00 | 19 | 0.00 | 79 | 0.00 |
| F83 Mixed specific developmental disorders | 5 | 0.00 | 7 | 0.00 | 5 | 0.00 | 1 | 0.00 | 7 | 0.00 | 3 | 0.00 | 28 | 0.00 |
| F84 Pervasive developmental disorders | 2 | 0.00 | 3 | 0.00 | 10 | 0.00 | 1 | 0.00 | 17 | 0.00 | 37 | 0.01 | 70 | 0.00 |
| F840 Childhood autism | 7 | 0.00 | 11 | 0.00 | 8 | 0.00 | 11 | 0.01 | 33 | 0.01 | 35 | 0.01 | 105 | 0.00 |
| F841 Atypical autism | 9 | 0.00 | 6 | 0.00 | 8 | 0.00 | 6 | 0.00 | 6 | 0.00 | 14 | 0.00 | 49 | 0.00 |
| F842 Retts syndrome | 1 | 0.00 |  | 0.00 |  | 0.00 |  | 0.00 | 1 | 0.00 | 7 | 0.00 | 9 | 0.00 |
| F843 Other childhood disintegrative disorder | 3 | 0.00 | 3 | 0.00 | 1 | 0.00 | 1 | 0.00 | 2 | 0.00 | 3 | 0.00 | 13 | 0.00 |
| F844 Overactive disorder associated with mental retardation and stereotyped | 1 | 0.00 | 3 | 0.00 | 4 | 0.00 | 4 | 0.00 |  | 0.00 | 6 | 0.00 | 18 | 0.00 |
| F845 Aspergers syndrome | 2 | 0.00 | 1 | 0.00 | 7 | 0.00 | 1 | 0.00 | 4 | 0.00 | 5 | 0.00 | 20 | 0.00 |
| F848 Other pervasive developmental disorders |  | 0.00 | 2 | 0.00 | 1 | 0.00 |  | 0.00 | 1 | 0.00 | 12 | 0.00 | 16 | 0.00 |
| F849 Pervasive developmental disorder, unspecified | 2 | 0.00 | 2 | 0.00 | 3 | 0.00 | 1 | 0.00 | 4 | 0.00 | 23 | 0.00 | 35 | 0.00 |
| F88 Other disorders of psychological development | 67 | 0.02 | 54 | 0.01 | 57 | 0.01 | 14 | 0.01 | 39 | 0.01 | 110 | 0.02 | 341 | 0.01 |
| F89 Unspecified disorder of psychological development | 27 | 0.01 | 44 | 0.01 | 36 | 0.01 | 43 | 0.02 | 60 | 0.01 | 82 | 0.01 | 292 | 0.01 |
| F90 Hyperkinetic disorders | 21 | 0.01 | 30 | 0.01 | 31 | 0.00 | 6 | 0.00 | 19 | 0.00 | 26 | 0.00 | 133 | 0.00 |
| F900 Disturbance of activity and attention | 5 | 0.00 | 7 | 0.00 | 14 | 0.00 | 5 | 0.00 | 3 | 0.00 | 12 | 0.00 | 46 | 0.00 |
| F901 Hyperkinetic conduct disorder | 4 | 0.00 | 6 | 0.00 | 5 | 0.00 |  | 0.00 |  | 0.00 | 3 | 0.00 | 18 | 0.00 |
| F908 Other hyperkinetic disorders |  | 0.00 | 4 | 0.00 | 3 | 0.00 | 3 | 0.00 | 3 | 0.00 | 4 | 0.00 | 17 | 0.00 |
| F909 Hyperkinetic disorder, unspecified | 11 | 0.00 | 5 | 0.00 | 14 | 0.00 | 8 | 0.00 | 5 | 0.00 | 23 | 0.00 | 66 | 0.00 |
| F91 Conduct disorders | 3 | 0.00 | 17 | 0.00 | 1 | 0.00 | 1 | 0.00 | 1 | 0.00 | 8 | 0.00 | 31 | 0.00 |
| F910 Conduct disorder confined to the family context | 4 | 0.00 | 1 | 0.00 | 3 | 0.00 | 1 | 0.00 | 5 | 0.00 | 4 | 0.00 | 18 | 0.00 |
| F911 Unsocialized conduct disorder | 2 | 0.00 | 1 | 0.00 | 1 | 0.00 |  | 0.00 |  | 0.00 | 7 | 0.00 | 11 | 0.00 |
| F912 Socialized conduct disorder | 4 | 0.00 |  | 0.00 |  | 0.00 | 1 | 0.00 |  | 0.00 |  | 0.00 | 5 | 0.00 |
| F913 Oppositional defiant disorder |  | 0.00 | 1 | 0.00 | 2 | 0.00 |  | 0.00 | 4 | 0.00 | 2 | 0.00 | 9 | 0.00 |
| F918 Other conduct disorders | 2 | 0.00 |  | 0.00 | 1 | 0.00 | 2 | 0.00 |  | 0.00 | 1 | 0.00 | 6 | 0.00 |
| F919 Conduct disorder, unspecified | 2 | 0.00 | 7 | 0.00 | 16 | 0.00 | 14 | 0.01 | 4 | 0.00 | 2 | 0.00 | 45 | 0.00 |
| F92 Mixed disorders of conduct and emotions | 3 | 0.00 | 5 | 0.00 | 27 | 0.00 | 7 | 0.00 | 7 | 0.00 | 1 | 0.00 | 50 | 0.00 |
| F920 Depressive conduct disorder | 3 | 0.00 |  | 0.00 | 3 | 0.00 | 2 | 0.00 | 2 | 0.00 | 3 | 0.00 | 13 | 0.00 |
| F928 Other mixed disorders of conduct and emotions |  | 0.00 |  | 0.00 |  | 0.00 | 2 | 0.00 | 3 | 0.00 | 1 | 0.00 | 6 | 0.00 |
| F929 Mixed disorder of conduct and emotions, unspecified | 2 | 0.00 | 1 | 0.00 | 1 | 0.00 |  | 0.00 |  | 0.00 |  | 0.00 | 4 | 0.00 |
| F93 Emotional disorders with onset specific to childhood | 1 | 0.00 |  | 0.00 | 1 | 0.00 | 2 | 0.00 | 1 | 0.00 | 3 | 0.00 | 8 | 0.00 |
| F930 Separation anxiety disorder of childhood | 6 | 0.00 | 2 | 0.00 | 2 | 0.00 |  | 0.00 | 2 | 0.00 | 1 | 0.00 | 13 | 0.00 |
| F931 Phobic anxiety disorder of childhood | 6 | 0.00 | 1 | 0.00 | 2 | 0.00 |  | 0.00 | 3 | 0.00 | 1 | 0.00 | 13 | 0.00 |
| F932 Social anxiety disorder of childhood | 8 | 0.00 | 5 | 0.00 | 8 | 0.00 | 4 | 0.00 | 1 | 0.00 | 3 | 0.00 | 29 | 0.00 |
| F933 Sibling rivalry disorder | 5 | 0.00 | 9 | 0.00 |  | 0.00 | 2 | 0.00 | 31 | 0.01 | 3 | 0.00 | 50 | 0.00 |
| F938 Other childhood emotional disorders | 1 | 0.00 |  | 0.00 | 2 | 0.00 |  | 0.00 |  | 0.00 | 2 | 0.00 | 5 | 0.00 |
| F939 Childhood emotional disorder, unspecified | 4 | 0.00 | 1 | 0.00 | 1 | 0.00 | 1 | 0.00 |  | 0.00 | 2 | 0.00 | 9 | 0.00 |
| F94 Disorders of social functioning with onset specific to childhood and ad | 4 | 0.00 | 21 | 0.00 | 4 | 0.00 | 2 | 0.00 | 5 | 0.00 | 8 | 0.00 | 44 | 0.00 |
| F940 Elective mutism | 31 | 0.01 | 26 | 0.00 | 16 | 0.00 | 12 | 0.01 | 7 | 0.00 | 19 | 0.00 | 111 | 0.00 |
| F941 Reactive attachment disorder of childhood | 5 | 0.00 | 4 | 0.00 | 2 | 0.00 | 2 | 0.00 | 7 | 0.00 | 6 | 0.00 | 26 | 0.00 |
| F942 Disinhibited attachment disorder of childhood | 1 | 0.00 | 14 | 0.00 | 26 | 0.00 | 3 | 0.00 | 14 | 0.00 | 29 | 0.00 | 87 | 0.00 |
| F948 Other childhood disorders of social functioning | 1 | 0.00 | 5 | 0.00 |  | 0.00 | 1 | 0.00 |  | 0.00 |  | 0.00 | 7 | 0.00 |
| F949 Childhood disorder of social functioning, unspecified |  | 0.00 |  | 0.00 | 1 | 0.00 |  | 0.00 |  | 0.00 | 1 | 0.00 | 2 | 0.00 |
| F95 Tic disorders | 2 | 0.00 |  | 0.00 |  | 0.00 |  | 0.00 | 1 | 0.00 | 1 | 0.00 | 4 | 0.00 |
| F950 Transient tic disorder | 5 | 0.00 | 10 | 0.00 | 4 | 0.00 | 7 | 0.00 | 9 | 0.00 | 7 | 0.00 | 42 | 0.00 |
| F951 Chronic motor or vocal tic disorder | 1 | 0.00 | 6 | 0.00 | 3 | 0.00 |  | 0.00 | 4 | 0.00 | 5 | 0.00 | 19 | 0.00 |
| F952 Combined vocal and multiple motor tic disorder [de la Tourette] |  | 0.00 |  | 0.00 |  | 0.00 |  | 0.00 |  | 0.00 | 1 | 0.00 | 1 | 0.00 |
| F959 Tic disorder, unspecified |  | 0.00 | 1 | 0.00 |  | 0.00 |  | 0.00 | 1 | 0.00 | 1 | 0.00 | 3 | 0.00 |
| F98 Other behavioural and emotional disorders with onset usually occurring | 12 | 0.00 | 3 | 0.00 | 2 | 0.00 | 1 | 0.00 | 1 | 0.00 | 5 | 0.00 | 24 | 0.00 |
| F980 Nonorganic enuresis |  | 0.00 | 1 | 0.00 |  | 0.00 |  | 0.00 | 1 | 0.00 | 5 | 0.00 | 7 | 0.00 |
| F981 Nonorganic encopresis | 2 | 0.00 | 2 | 0.00 | 1 | 0.00 |  | 0.00 | 2 | 0.00 |  | 0.00 | 7 | 0.00 |
| F982 Feeding disorder of infancy and childhood | 20 | 0.01 | 7 | 0.00 | 10 | 0.00 | 6 | 0.00 | 14 | 0.00 | 67 | 0.01 | 124 | 0.00 |
| F983 Pica of infancy and childhood |  | 0.00 |  | 0.00 |  | 0.00 | 1 | 0.00 | 1 | 0.00 | 2 | 0.00 | 4 | 0.00 |
| F984 Stereotyped movement disorders |  | 0.00 | 2 | 0.00 | 1 | 0.00 | 4 | 0.00 | 9 | 0.00 | 16 | 0.00 | 32 | 0.00 |
| F985 Stuttering [stammering] | 1 | 0.00 | 2 | 0.00 | 2 | 0.00 | 2 | 0.00 | 1 | 0.00 | 2 | 0.00 | 10 | 0.00 |
| F986 Cluttering |  | 0.00 |  | 0.00 |  | 0.00 |  | 0.00 | 2 | 0.00 | 2 | 0.00 | 4 | 0.00 |
| F988 Other specified behavioural and emotional disorders with onset usually | 1 | 0.00 | 1 | 0.00 |  | 0.00 |  | 0.00 | 1 | 0.00 | 2 | 0.00 | 5 | 0.00 |
| F989 Unspecified behavioural and emotional disorders with onset usually occ | 22 | 0.01 | 22 | 0.00 | 20 | 0.00 | 3 | 0.00 | 9 | 0.00 | 15 | 0.00 | 91 | 0.00 |
| F99 Mental disorder, not otherwise specified | 1696 | 0.47 | 1708 | 0.32 | 1959 | 0.30 | 1034 | 0.51 | 1101 | 0.21 | 1358 | 0.23 | 8856 | 0.31 |
| J09 Influenza due to identified avian influenza virus | 160 | 0.04 | 70 | 0.01 | 116 | 0.02 | 33 | 0.02 | 32 | 0.01 | 71 | 0.01 | 482 | 0.02 |
| J10 Influenza due to identified influenza virus | 334 | 0.09 | 437 | 0.08 | 415 | 0.06 | 187 | 0.09 | 218 | 0.04 | 347 | 0.06 | 1938 | 0.07 |
| J100 Influenza with pneumonia, influenza virus identified | 438 | 0.12 | 374 | 0.07 | 682 | 0.11 | 276 | 0.14 | 685 | 0.13 | 265 | 0.05 | 2720 | 0.09 |
| J101 Influenza with other respiratory manifestations, influenza virus ident | 15 | 0.00 | 12 | 0.00 | 10 | 0.00 | 4 | 0.00 | 7 | 0.00 | 4 | 0.00 | 52 | 0.00 |
| J108 Influenza with other manifestations, influenza virus identified | 4 | 0.00 | 7 | 0.00 | 9 | 0.00 |  | 0.00 | 6 | 0.00 | 5 | 0.00 | 31 | 0.00 |
| J11 Influenza, virus not identified | 907 | 0.25 | 890 | 0.16 | 1140 | 0.18 | 268 | 0.13 | 390 | 0.07 | 352 | 0.06 | 3947 | 0.14 |
| J110 Influenza with pneumonia, virus not identified | 579 | 0.16 | 518 | 0.10 | 591 | 0.09 | 289 | 0.14 | 793 | 0.15 | 498 | 0.08 | 3268 | 0.11 |
| J111 Influenza with other respiratory manifestations, virus not identified | 57 | 0.02 | 140 | 0.03 | 361 | 0.06 | 7 | 0.00 | 31 | 0.01 | 50 | 0.01 | 646 | 0.02 |
| J118 Influenza with other manifestations, virus not identified | 14 | 0.00 | 8 | 0.00 | 18 | 0.00 | 3 | 0.00 | 4 | 0.00 | 10 | 0.00 | 57 | 0.00 |
| J12 Viral pneumonia, NEC | 3980 | 1.11 | 4154 | 0.77 | 5639 | 0.87 | 2108 | 1.05 | 4950 | 0.93 | 3836 | 0.65 | 24667 | 0.86 |
| J120 Adenoviral pneumonia | 193 | 0.05 | 141 | 0.03 | 127 | 0.02 | 27 | 0.01 | 332 | 0.06 | 381 | 0.06 | 1201 | 0.04 |
| J121 Respiratory syncytial virus pneumonia | 422 | 0.12 | 376 | 0.07 | 328 | 0.05 | 211 | 0.10 | 358 | 0.07 | 450 | 0.08 | 2145 | 0.07 |
| J122 Parainfluenza virus pneumonia | 37 | 0.01 | 27 | 0.00 | 46 | 0.01 | 7 | 0.00 | 13 | 0.00 | 30 | 0.01 | 160 | 0.01 |
| J123 Human metapneumovirus pneumonia | 10 | 0.00 | 6 | 0.00 | 7 | 0.00 | 2 | 0.00 | 3 | 0.00 | 7 | 0.00 | 35 | 0.00 |
| J128 Other viral pneumonia | 2071 | 0.58 | 1589 | 0.29 | 1371 | 0.21 | 622 | 0.31 | 1446 | 0.27 | 2312 | 0.39 | 9411 | 0.33 |
| J129 Viral pneumonia, unspecified | 3516 | 0.98 | 3767 | 0.70 | 3791 | 0.59 | 1065 | 0.53 | 2739 | 0.52 | 1438 | 0.24 | 16316 | 0.57 |
| J13 Pneumonia due to Streptococcus pneumoniae | 1443 | 0.40 | 1362 | 0.25 | 1195 | 0.19 | 167 | 0.08 | 228 | 0.04 | 931 | 0.16 | 5326 | 0.19 |
| J14 Pneumonia due to Haemophilus influenzae | 467 | 0.13 | 685 | 0.13 | 828 | 0.13 | 375 | 0.19 | 657 | 0.12 | 1042 | 0.18 | 4054 | 0.14 |
| J15 Bacterial pneumonia, NEC | 2224 | 0.62 | 2247 | 0.42 | 3223 | 0.50 | 1473 | 0.73 | 2064 | 0.39 | 4055 | 0.69 | 15286 | 0.53 |
| J150 Pneumonia due to Klebsiella pneumoniae | 59 | 0.02 | 286 | 0.05 | 798 | 0.12 | 37 | 0.02 | 39 | 0.01 | 798 | 0.14 | 2017 | 0.07 |
| J151 Pneumonia due to Pseudomonas | 53 | 0.01 | 81 | 0.01 | 352 | 0.05 | 13 | 0.01 | 21 | 0.00 | 220 | 0.04 | 740 | 0.03 |
| J152 Pneumonia due to staphylococcus | 339 | 0.09 | 385 | 0.07 | 341 | 0.05 | 14 | 0.01 | 27 | 0.01 | 51 | 0.01 | 1157 | 0.04 |
| J153 Pneumonia due to streptococcus, group B | 44 | 0.01 | 59 | 0.01 | 31 | 0.00 | 1 | 0.00 | 17 | 0.00 | 22 | 0.00 | 174 | 0.01 |
| J154 Pneumonia due to other streptococci | 72 | 0.02 | 91 | 0.02 | 22 | 0.00 | 6 | 0.00 | 211 | 0.04 | 77 | 0.01 | 479 | 0.02 |
| J155 Pneumonia due to Escherichia coli | 13 | 0.00 | 5 | 0.00 | 6 | 0.00 | 3 | 0.00 | 7 | 0.00 | 10 | 0.00 | 44 | 0.00 |
| J156 Pneumonia due to other aerobic Gram-negative bacteria | 11 | 0.00 | 296 | 0.05 | 3 | 0.00 | 9 | 0.00 | 23 | 0.00 | 12 | 0.00 | 354 | 0.01 |
| J157 Pneumonia due to Mycoplasma pneumoniae | 23 | 0.01 | 828 | 0.15 | 18 | 0.00 | 7 | 0.00 | 3 | 0.00 | 7 | 0.00 | 886 | 0.03 |
| J158 Other bacterial pneumonia | 446 | 0.12 | 246 | 0.05 | 168 | 0.03 | 74 | 0.04 | 185 | 0.03 | 506 | 0.09 | 1625 | 0.06 |
| J159 Bacterial pneumonia, unspecified | 2099 | 0.58 | 2993 | 0.55 | 3111 | 0.48 | 586 | 0.29 | 1265 | 0.24 | 2215 | 0.38 | 12269 | 0.43 |
| J17 Pneumonia in diseases classified elsewhere | 317 | 0.09 | 520 | 0.10 | 581 | 0.09 | 337 | 0.17 | 545 | 0.10 | 932 | 0.16 | 3232 | 0.11 |
| J170 Pneumonia in bacterial diseases classified elsewhere | 13 | 0.00 | 14 | 0.00 | 17 | 0.00 | 5 | 0.00 | 10 | 0.00 | 8 | 0.00 | 67 | 0.00 |
| J171 Pneumonia in viral diseases classified elsewhere | 8 | 0.00 | 3 | 0.00 | 10 | 0.00 | 6 | 0.00 | 10 | 0.00 | 19 | 0.00 | 56 | 0.00 |
| J172 Pneumonia in mycoses | 12 | 0.00 | 8 | 0.00 | 2 | 0.00 | 3 | 0.00 | 10 | 0.00 | 16 | 0.00 | 51 | 0.00 |
| J173 Pneumonia in parasitic diseases | 2 | 0.00 | 9 | 0.00 | 9 | 0.00 |  | 0.00 | 5 | 0.00 | 5 | 0.00 | 30 | 0.00 |
| J178 Pneumonia in other diseases classified elsewhere | 8 | 0.00 | 13 | 0.00 | 14 | 0.00 | 10 | 0.00 | 19 | 0.00 | 31 | 0.01 | 95 | 0.00 |
| J18 Pneumonia, organism unspecified | 34275 | 9.55 | 39460 | 7.29 | 57042 | 8.85 | 22269 | 11.04 | 38625 | 7.28 | 35054 | 5.96 | 226725 | 7.91 |
| J180 Bronchopneumonia, unspecified | 10278 | 2.86 | 12694 | 2.35 | 17371 | 2.69 | 8402 | 4.17 | 17312 | 3.26 | 29632 | 5.03 | 95689 | 3.34 |
| J181 Lobar pneumonia, unspecified | 226 | 0.06 | 187 | 0.03 | 309 | 0.05 | 210 | 0.10 | 413 | 0.08 | 321 | 0.05 | 1666 | 0.06 |
| J182 Hypostatic pneumonia, unspecified | 33 | 0.01 | 17 | 0.00 | 35 | 0.01 | 26 | 0.01 | 19 | 0.00 | 25 | 0.00 | 155 | 0.01 |
| J188 Other pneumonia, organism unspecified | 398 | 0.11 | 858 | 0.16 | 584 | 0.09 | 193 | 0.10 | 173 | 0.03 | 509 | 0.09 | 2715 | 0.09 |
| J189 Pneumonia, unspecified | 32603 | 9.08 | 35807 | 6.62 | 46945 | 7.28 | 19263 | 9.55 | 38879 | 7.33 | 40040 | 6.80 | 213537 | 7.45 |
| L20 Atopic dermatitis | 31 | 0.01 | 45 | 0.01 | 39 | 0.01 | 25 | 0.01 | 17 | 0.00 | 52 | 0.01 | 209 | 0.01 |
| L200 Besniers prurigo | 1 | 0.00 |  | 0.00 |  | 0.00 |  | 0.00 | 2 | 0.00 | 2 | 0.00 | 5 | 0.00 |
| L208 Other atopic dermatitis | 5 | 0.00 | 9 | 0.00 | 9 | 0.00 | 3 | 0.00 | 6 | 0.00 | 13 | 0.00 | 45 | 0.00 |
| L209 Atopic dermatitis, unspecified | 20 | 0.01 | 5 | 0.00 | 18 | 0.00 | 5 | 0.00 | 7 | 0.00 | 12 | 0.00 | 67 | 0.00 |
| L21 Seborrhoeic dermatitis | 13 | 0.00 | 19 | 0.00 | 10 | 0.00 | 6 | 0.00 | 10 | 0.00 | 27 | 0.00 | 85 | 0.00 |
| L210 Seborrhoea capitis | 2 | 0.00 | 3 | 0.00 | 2 | 0.00 | 2 | 0.00 | 2 | 0.00 | 5 | 0.00 | 16 | 0.00 |
| L211 Seborrhoeic infantile dermatitis | 3 | 0.00 | 2 | 0.00 |  | 0.00 | 1 | 0.00 |  | 0.00 |  | 0.00 | 6 | 0.00 |
| L218 Other seborrhoeic dermatitis | 19 | 0.01 | 6 | 0.00 | 6 | 0.00 | 1 | 0.00 | 2 | 0.00 | 6 | 0.00 | 40 | 0.00 |
| L219 Seborrhoeic dermatitis, unspecified | 4 | 0.00 | 3 | 0.00 | 2 | 0.00 | 1 | 0.00 | 6 | 0.00 | 6 | 0.00 | 22 | 0.00 |
| L23 Allergic contact dermatitis | 75 | 0.02 | 67 | 0.01 | 120 | 0.02 | 64 | 0.03 | 59 | 0.01 | 131 | 0.02 | 516 | 0.02 |
| L230 Allergic contact dermatitis due to metals | 2 | 0.00 | 4 | 0.00 | 7 | 0.00 | 4 | 0.00 |  | 0.00 | 3 | 0.00 | 20 | 0.00 |
| L231 Allergic contact dermatitis due to adhesives | 3 | 0.00 | 2 | 0.00 | 3 | 0.00 | 3 | 0.00 | 1 | 0.00 | 2 | 0.00 | 14 | 0.00 |
| L232 Allergic contact dermatitis due to cosmetics | 5 | 0.00 | 2 | 0.00 | 1 | 0.00 | 1 | 0.00 | 2 | 0.00 | 3 | 0.00 | 14 | 0.00 |
| L233 Allergic contact dermatitis due to drugs in contact with skin | 11 | 0.00 | 8 | 0.00 | 21 | 0.00 | 6 | 0.00 | 10 | 0.00 | 12 | 0.00 | 68 | 0.00 |
| L234 Allergic contact dermatitis due to dyes | 9 | 0.00 | 3 | 0.00 | 4 | 0.00 | 1 | 0.00 | 2 | 0.00 | 6 | 0.00 | 25 | 0.00 |
| L235 Allergic contact dermatitis due to other chemical products | 2 | 0.00 | 8 | 0.00 | 8 | 0.00 | 5 | 0.00 | 2 | 0.00 | 7 | 0.00 | 32 | 0.00 |
| L236 Allergic contact dermatitis due to food in contact with skin | 7 | 0.00 | 10 | 0.00 | 10 | 0.00 | 8 | 0.00 | 1 | 0.00 | 18 | 0.00 | 54 | 0.00 |
| L237 Allergic contact dermatitis due to plants, except food |  | 0.00 | 3 | 0.00 | 8 | 0.00 | 6 | 0.00 | 2 | 0.00 | 7 | 0.00 | 26 | 0.00 |
| L238 Allergic contact dermatitis due to other agents | 3 | 0.00 | 1 | 0.00 | 5 | 0.00 | 7 | 0.00 | 4 | 0.00 | 4 | 0.00 | 24 | 0.00 |
| L239 Allergic contact dermatitis, unspecified cause | 26 | 0.01 | 22 | 0.00 | 29 | 0.00 | 21 | 0.01 | 9 | 0.00 | 12 | 0.00 | 119 | 0.00 |
| L24 Irritant contact dermatitis | 6 | 0.00 | 5 | 0.00 | 3 | 0.00 | 2 | 0.00 | 2 | 0.00 | 20 | 0.00 | 38 | 0.00 |
| L240 Irritant contact dermatitis due to detergents |  | 0.00 | 1 | 0.00 | 1 | 0.00 | 1 | 0.00 | 1 | 0.00 | 2 | 0.00 | 6 | 0.00 |
| L241 Irritant contact dermatitis due to oils and greases |  | 0.00 | 1 | 0.00 |  | 0.00 |  | 0.00 |  | 0.00 |  | 0.00 | 1 | 0.00 |
| L242 Irritant contact dermatitis due to solvents |  | 0.00 |  | 0.00 |  | 0.00 |  | 0.00 | 1 | 0.00 | 3 | 0.00 | 4 | 0.00 |
| L243 Irritant contact dermatitis due to cosmetics | 1 | 0.00 | 1 | 0.00 |  | 0.00 |  | 0.00 | 1 | 0.00 | 3 | 0.00 | 6 | 0.00 |
| L244 Irritant contact dermatitis due to drugs in contact with skin |  | 0.00 | 1 | 0.00 |  | 0.00 | 1 | 0.00 |  | 0.00 |  | 0.00 | 2 | 0.00 |
| L245 Irritant contact dermatitis due to other chemical products | 2 | 0.00 | 1 | 0.00 |  | 0.00 | 2 | 0.00 | 1 | 0.00 | 3 | 0.00 | 9 | 0.00 |
| L246 Irritant contact dermatitis due to food in contact with skin | 15 | 0.00 | 13 | 0.00 | 16 | 0.00 | 7 | 0.00 | 3 | 0.00 | 2 | 0.00 | 56 | 0.00 |
| L247 Irritant contact dermatitis due to plants, except food | 2 | 0.00 |  | 0.00 |  | 0.00 |  | 0.00 |  | 0.00 | 7 | 0.00 | 9 | 0.00 |
| L249 Irritant contact dermatitis, unspecified cause | 2 | 0.00 | 1 | 0.00 | 3 | 0.00 | 2 | 0.00 | 1 | 0.00 | 3 | 0.00 | 12 | 0.00 |
| L25 Unspecified contact dermatitis | 3 | 0.00 | 16 | 0.00 | 7 | 0.00 | 12 | 0.01 | 12 | 0.00 | 14 | 0.00 | 64 | 0.00 |
| L250 Unspecified contact dermatitis due to cosmetics | 2 | 0.00 | 3 | 0.00 | 2 | 0.00 | 2 | 0.00 | 1 | 0.00 | 1 | 0.00 | 11 | 0.00 |
| L251 Unspecified contact dermatitis due to drugs in contact with skin | 26 | 0.01 | 19 | 0.00 | 2 | 0.00 | 3 | 0.00 | 3 | 0.00 | 1 | 0.00 | 54 | 0.00 |
| L252 Unspecified contact dermatitis due to dyes | 1 | 0.00 | 3 | 0.00 |  | 0.00 | 1 | 0.00 |  | 0.00 |  | 0.00 | 5 | 0.00 |
| L253 Unspecified contact dermatitis due to other chemical products | 6 | 0.00 | 3 | 0.00 | 5 | 0.00 | 3 | 0.00 | 1 | 0.00 | 4 | 0.00 | 22 | 0.00 |
| L254 Unspecified contact dermatitis due to food in contact with skin | 1 | 0.00 | 2 | 0.00 | 5 | 0.00 |  | 0.00 | 1 | 0.00 | 7 | 0.00 | 16 | 0.00 |
| L255 Unspecified contact dermatitis due to plants, except food | 5 | 0.00 | 1 | 0.00 | 21 | 0.00 | 58 | 0.03 | 40 | 0.01 | 7 | 0.00 | 132 | 0.00 |
| L258 Unspecified contact dermatitis due to other agents | 2 | 0.00 | 1 | 0.00 |  | 0.00 |  | 0.00 |  | 0.00 | 1 | 0.00 | 4 | 0.00 |
| L259 Unspecified contact dermatitis, unspecified cause | 1 | 0.00 | 2 | 0.00 | 4 | 0.00 | 2 | 0.00 | 3 | 0.00 |  | 0.00 | 12 | 0.00 |
| L26 Exfoliative dermatitis | 4 | 0.00 | 8 | 0.00 | 7 | 0.00 | 2 | 0.00 | 8 | 0.00 | 34 | 0.01 | 63 | 0.00 |
| L27 Dermatitis due to substances taken internally | 4 | 0.00 | 4 | 0.00 | 5 | 0.00 | 4 | 0.00 | 2 | 0.00 | 14 | 0.00 | 33 | 0.00 |
| L270 Generalized skin eruption due to drugs and medicaments | 4 | 0.00 | 6 | 0.00 | 11 | 0.00 | 5 | 0.00 | 8 | 0.00 | 19 | 0.00 | 53 | 0.00 |
| L271 Localized skin eruption due to drugs and medicaments | 1 | 0.00 |  | 0.00 |  | 0.00 | 1 | 0.00 | 1 | 0.00 | 3 | 0.00 | 6 | 0.00 |
| L272 Dermatitis due to ingested food | 5 | 0.00 | 5 | 0.00 | 7 | 0.00 | 2 | 0.00 | 3 | 0.00 | 4 | 0.00 | 26 | 0.00 |
| L278 Dermatitis due to other substances taken internally |  | 0.00 |  | 0.00 |  | 0.00 | 1 | 0.00 |  | 0.00 | 1 | 0.00 | 2 | 0.00 |
| L279 Dermatitis due to unspecified substance taken internally | 1 | 0.00 | 1 | 0.00 |  | 0.00 |  | 0.00 | 2 | 0.00 | 2 | 0.00 | 6 | 0.00 |
| L28 Lichen simplex chronicus and prurigo | 1 | 0.00 | 1 | 0.00 | 2 | 0.00 |  | 0.00 | 3 | 0.00 | 10 | 0.00 | 17 | 0.00 |
| L280 Lichen simplex chronicus |  | 0.00 |  | 0.00 | 2 | 0.00 | 1 | 0.00 | 1 | 0.00 |  | 0.00 | 4 | 0.00 |
| L281 Prurigo nodularis | 1 | 0.00 | 2 | 0.00 | 3 | 0.00 | 1 | 0.00 |  | 0.00 | 1 | 0.00 | 8 | 0.00 |
| L282 Other prurigo |  | 0.00 |  | 0.00 | 1 | 0.00 |  | 0.00 |  | 0.00 | 2 | 0.00 | 3 | 0.00 |
| L29 Pruritus | 7 | 0.00 | 4 | 0.00 | 12 | 0.00 | 4 | 0.00 | 8 | 0.00 | 26 | 0.00 | 61 | 0.00 |
| L290 Pruritus ani |  | 0.00 |  | 0.00 | 1 | 0.00 | 1 | 0.00 |  | 0.00 |  | 0.00 | 2 | 0.00 |
| L291 Pruritus scroti | 1 | 0.00 | 4 | 0.00 | 3 | 0.00 |  | 0.00 | 3 | 0.00 | 1 | 0.00 | 12 | 0.00 |
| L292 Pruritus vulvae | 4 | 0.00 | 3 | 0.00 | 4 | 0.00 |  | 0.00 | 3 | 0.00 | 1 | 0.00 | 15 | 0.00 |
| L293 Anogenital pruritus, unspecified |  | 0.00 | 1 | 0.00 |  | 0.00 |  | 0.00 | 1 | 0.00 | 1 | 0.00 | 3 | 0.00 |
| L298 Other pruritus |  | 0.00 |  | 0.00 |  | 0.00 | 2 | 0.00 | 1 | 0.00 | 1 | 0.00 | 4 | 0.00 |
| L299 Pruritus, unspecified | 1 | 0.00 | 3 | 0.00 | 6 | 0.00 | 4 | 0.00 |  | 0.00 | 14 | 0.00 | 28 | 0.00 |
| L30 Other dermatitis | 9 | 0.00 | 22 | 0.00 | 22 | 0.00 | 13 | 0.01 | 19 | 0.00 | 36 | 0.01 | 121 | 0.00 |
| L300 Nummular dermatitis | 1 | 0.00 | 3 | 0.00 | 1 | 0.00 |  | 0.00 |  | 0.00 |  | 0.00 | 5 | 0.00 |
| L301 Dyshidrosis [pompholyx] | 3 | 0.00 | 1 | 0.00 | 1 | 0.00 | 4 | 0.00 | 1 | 0.00 | 3 | 0.00 | 13 | 0.00 |
| L302 Cutaneous autosensitization |  | 0.00 |  | 0.00 |  | 0.00 | 2 | 0.00 |  | 0.00 | 4 | 0.00 | 6 | 0.00 |
| L303 Infective dermatitis | 3 | 0.00 | 8 | 0.00 | 2 | 0.00 | 1 | 0.00 | 3 | 0.00 | 9 | 0.00 | 26 | 0.00 |
| L304 Erythema intertrigo | 1 | 0.00 | 5 | 0.00 | 5 | 0.00 | 1 | 0.00 | 2 | 0.00 | 6 | 0.00 | 20 | 0.00 |
| L305 Pityriasis alba |  | 0.00 |  | 0.00 |  | 0.00 |  | 0.00 | 1 | 0.00 | 1 | 0.00 | 2 | 0.00 |
| L308 Other specified dermatitis |  | 0.00 | 1 | 0.00 | 1 | 0.00 |  | 0.00 | 1 | 0.00 | 1 | 0.00 | 4 | 0.00 |
| L309 Dermatitis, unspecified | 8 | 0.00 | 10 | 0.00 | 24 | 0.00 | 13 | 0.01 | 6 | 0.00 | 14 | 0.00 | 75 | 0.00 |
| N39 Other disorders of urinary system | 2391 | 0.67 | 2926 | 0.54 | 4054 | 0.63 | 2553 | 1.27 | 4018 | 0.76 | 6663 | 1.13 | 22605 | 0.79 |
| N390 Urinary tract infection, site not specified | 24891 | 6.94 | 38150 | 7.05 | 47411 | 7.36 | 28104 | 13.93 | 32432 | 6.11 | 49579 | 8.42 | 220567 | 7.70 |
| N391 Persistent proteinuria, unspecified | 20 | 0.01 | 25 | 0.00 | 31 | 0.00 | 25 | 0.01 | 16 | 0.00 | 22 | 0.00 | 139 | 0.00 |
| N392 Orthostatic proteinuria, unspecified | 4 | 0.00 | 13 | 0.00 | 13 | 0.00 | 8 | 0.00 | 10 | 0.00 | 34 | 0.01 | 82 | 0.00 |
| N393 Stress incontinence | 25 | 0.01 | 45 | 0.01 | 47 | 0.01 | 37 | 0.02 | 39 | 0.01 | 58 | 0.01 | 251 | 0.01 |
| N394 Other specified urinary incontinence | 259 | 0.07 | 699 | 0.13 | 1147 | 0.18 | 298 | 0.15 | 398 | 0.08 | 300 | 0.05 | 3101 | 0.11 |
| N398 Other specified disorders of urinary system | 34 | 0.01 | 34 | 0.01 | 25 | 0.00 | 63 | 0.03 | 109 | 0.02 | 55 | 0.01 | 320 | 0.01 |
| N399 Disorder of urinary system, unspecified | 136 | 0.04 | 207 | 0.04 | 152 | 0.02 | 146 | 0.07 | 82 | 0.02 | 179 | 0.03 | 902 | 0.03 |
| N46 Male infertility |  | 0.00 | 1 | 0.00 | 4 | 0.00 | 2 | 0.00 | 3 | 0.00 | 30 | 0.01 | 40 | 0.00 |
| N97 Female infertility | 43 | 0.01 | 76 | 0.01 | 53 | 0.01 | 44 | 0.02 | 84 | 0.02 | 243 | 0.04 | 543 | 0.02 |
| N970 Female infertility associated with anovulation | 2 | 0.00 |  | 0.00 | 10 | 0.00 | 7 | 0.00 | 6 | 0.00 | 18 | 0.00 | 43 | 0.00 |
| N971 Female infertility of tubal origin | 6 | 0.00 | 6 | 0.00 | 6 | 0.00 | 5 | 0.00 | 14 | 0.00 | 67 | 0.01 | 104 | 0.00 |
| N972 Female infertility of uterine origin | 4 | 0.00 | 14 | 0.00 | 11 | 0.00 | 4 | 0.00 | 2 | 0.00 | 9 | 0.00 | 44 | 0.00 |
| N973 Female infertility of cervical origin | 1 | 0.00 | 4 | 0.00 | 3 | 0.00 | 4 | 0.00 | 5 | 0.00 | 11 | 0.00 | 28 | 0.00 |
| N974 Female infertility associated with male factors | 1 | 0.00 |  | 0.00 |  | 0.00 | 2 | 0.00 |  | 0.00 | 1 | 0.00 | 4 | 0.00 |
| N978 Female infertility of other origin | 2 | 0.00 | 1 | 0.00 | 1 | 0.00 |  | 0.00 | 1 | 0.00 | 8 | 0.00 | 13 | 0.00 |
| N979 Female infertility, unspecified | 10 | 0.00 | 6 | 0.00 | 9 | 0.00 | 12 | 0.01 | 18 | 0.00 | 61 | 0.01 | 116 | 0.00 |
| O12 Gestational [pregnancy-induced] oedema and proteinuria without hyperten | 530 | 0.15 | 505 | 0.09 | 597 | 0.09 | 362 | 0.18 | 414 | 0.08 | 663 | 0.11 | 3071 | 0.11 |
| O120 Gestational oedema | 17 | 0.00 | 56 | 0.01 | 67 | 0.01 | 50 | 0.02 | 33 | 0.01 | 42 | 0.01 | 265 | 0.01 |
| O121 Gestational proteinuria | 3 | 0.00 | 20 | 0.00 | 7 | 0.00 |  | 0.00 | 6 | 0.00 | 4 | 0.00 | 40 | 0.00 |
| O122 Gestational oedema with proteinuria | 4 | 0.00 | 8 | 0.00 | 11 | 0.00 | 10 | 0.00 | 5 | 0.00 | 10 | 0.00 | 48 | 0.00 |
| O13 Gestational [pregnancy-induced] hypertension without significant protei | 115 | 0.03 | 74 | 0.01 | 120 | 0.02 | 55 | 0.03 | 77 | 0.01 | 198 | 0.03 | 639 | 0.02 |
| O14 Gestational [pregnancy-induced] hypertension with significant proteinur | 255 | 0.07 | 202 | 0.04 | 576 | 0.09 | 80 | 0.04 | 126 | 0.02 | 517 | 0.09 | 1756 | 0.06 |
| O140 Moderate pre-eclampsia | 116 | 0.03 | 119 | 0.02 | 158 | 0.02 | 211 | 0.10 | 206 | 0.04 | 292 | 0.05 | 1102 | 0.04 |
| O141 Severe pre-eclampsia | 316 | 0.09 | 474 | 0.09 | 647 | 0.10 | 502 | 0.25 | 662 | 0.12 | 789 | 0.13 | 3390 | 0.12 |
| O149 Pre-eclampsia, unspecified | 809 | 0.23 | 1402 | 0.26 | 1504 | 0.23 | 1213 | 0.60 | 1183 | 0.22 | 1743 | 0.30 | 7854 | 0.27 |
| O15 Eclampsia | 953 | 0.27 | 1615 | 0.30 | 1713 | 0.27 | 1480 | 0.73 | 1448 | 0.27 | 2114 | 0.36 | 9323 | 0.33 |
| O150 Eclampsia in pregnancy | 581 | 0.16 | 596 | 0.11 | 827 | 0.13 | 972 | 0.48 | 1074 | 0.20 | 1234 | 0.21 | 5284 | 0.18 |
| O151 Eclampsia in labour | 325 | 0.09 | 2226 | 0.41 | 322 | 0.05 | 497 | 0.25 | 215 | 0.04 | 396 | 0.07 | 3981 | 0.14 |
| O152 Eclampsia in the puerperium | 327 | 0.09 | 439 | 0.08 | 496 | 0.08 | 435 | 0.22 | 722 | 0.14 | 876 | 0.15 | 3295 | 0.11 |
| O159 Eclampsia, unspecified as to time period | 256 | 0.07 | 369 | 0.07 | 314 | 0.05 | 304 | 0.15 | 227 | 0.04 | 303 | 0.05 | 1773 | 0.06 |
| T57 Toxic effect of other inorganic substances | 40 | 0.01 | 21 | 0.00 | 8 | 0.00 | 3 | 0.00 | 8 | 0.00 | 22 | 0.00 | 102 | 0.00 |
| T570 Arsenic and its compounds |  | 0.00 | 16 | 0.00 | 3 | 0.00 | 5 | 0.00 | 1 | 0.00 | 2 | 0.00 | 27 | 0.00 |
| T571 Phosphorus and its compounds | 274 | 0.08 | 387 | 0.07 | 328 | 0.05 | 278 | 0.14 | 266 | 0.05 | 270 | 0.05 | 1803 | 0.06 |
| T572 Manganese and its compounds | 1 | 0.00 |  | 0.00 |  | 0.00 |  | 0.00 |  | 0.00 |  | 0.00 | 1 | 0.00 |
| T573 Hydrogen cyanide |  | 0.00 | 2 | 0.00 |  | 0.00 |  | 0.00 | 3 | 0.00 | 3 | 0.00 | 8 | 0.00 |
| T578 Other specified inorganic substances |  | 0.00 | 1 | 0.00 | 1 | 0.00 |  | 0.00 | 1 | 0.00 |  | 0.00 | 3 | 0.00 |
| T579 Inorganic substance, unspecified | 7 | 0.00 | 5 | 0.00 | 1 | 0.00 |  | 0.00 | 3 | 0.00 | 6 | 0.00 | 22 | 0.00 |
| T63 Toxic effect of contact with venomous animals | 213 | 0.06 | 541 | 0.10 | 669 | 0.10 | 408 | 0.20 | 594 | 0.11 | 2461 | 0.42 | 4886 | 0.17 |
| T630 Snake venom | 1880 | 0.52 | 2763 | 0.51 | 2635 | 0.41 | 2383 | 1.18 | 3250 | 0.61 | 8118 | 1.38 | 21029 | 0.73 |
| T631 Venom of other reptiles | 6 | 0.00 | 23 | 0.00 | 26 | 0.00 | 19 | 0.01 | 10 | 0.00 | 36 | 0.01 | 120 | 0.00 |
| T632 Venom of scorpion | 5 | 0.00 | 5 | 0.00 | 9 | 0.00 | 12 | 0.01 | 32 | 0.01 | 38 | 0.01 | 101 | 0.00 |
| T633 Venom of spider | 63 | 0.02 | 76 | 0.01 | 141 | 0.02 | 44 | 0.02 | 34 | 0.01 | 40 | 0.01 | 398 | 0.01 |
| T634 Venom of other arthropods | 434 | 0.12 | 456 | 0.08 | 749 | 0.12 | 407 | 0.20 | 401 | 0.08 | 709 | 0.12 | 3156 | 0.11 |
| T635 Toxic effect of contact with fish | 12 | 0.00 | 11 | 0.00 | 24 | 0.00 | 12 | 0.01 | 9 | 0.00 | 13 | 0.00 | 81 | 0.00 |
| T636 Toxic effect of contact with other marine animals | 2 | 0.00 | 10 | 0.00 | 7 | 0.00 | 10 | 0.00 | 4 | 0.00 | 15 | 0.00 | 48 | 0.00 |
| T638 Toxic effect of contact with other venomous animals | 6 | 0.00 | 4 | 0.00 | 14 | 0.00 | 7 | 0.00 | 4 | 0.00 | 11 | 0.00 | 46 | 0.00 |
| T639 Toxic effect of contact with unspecified venomous animal | 28 | 0.01 | 14 | 0.00 | 14 | 0.00 | 35 | 0.02 | 28 | 0.01 | 113 | 0.02 | 232 | 0.01 |
| W69 Drowning and submersion while in natural water | 35 | 0.01 | 52 | 0.01 | 80 | 0.01 | 129 | 0.06 | 97 | 0.02 | 122 | 0.02 | 515 | 0.02 |
| W690 Drowning and submersion while in natural water, home | 5 | 0.00 | 34 | 0.01 | 54 | 0.01 | 52 | 0.03 | 33 | 0.01 | 26 | 0.00 | 204 | 0.01 |
| W691 Drowning and submersion while in natural water, residential institutio |  | 0.00 | 2 | 0.00 | 1 | 0.00 | 1 | 0.00 | 2 | 0.00 |  | 0.00 | 6 | 0.00 |
| W692 Drowning and submersion while in natural water, school, other institut | 1 | 0.00 |  | 0.00 |  | 0.00 |  | 0.00 | 2 | 0.00 |  | 0.00 | 3 | 0.00 |
| W693 Drowning and submersion while in natural water, sports and athletics a |  | 0.00 |  | 0.00 |  | 0.00 | 1 | 0.00 | 3 | 0.00 |  | 0.00 | 4 | 0.00 |
| W694 Drowning and submersion while in natural water, street and highway |  | 0.00 | 1 | 0.00 | 10 | 0.00 | 1 | 0.00 |  | 0.00 |  | 0.00 | 12 | 0.00 |
| W695 Drowning and submersion while in natural water, trade and service area | 1 | 0.00 | 2 | 0.00 |  | 0.00 |  | 0.00 |  | 0.00 |  | 0.00 | 3 | 0.00 |
| W696 Drowning and submersion while in natural water, industrial and constru |  | 0.00 | 1 | 0.00 |  | 0.00 |  | 0.00 | 1 | 0.00 |  | 0.00 | 2 | 0.00 |
| W697 Drowning and submersion while in natural water, farm |  | 0.00 | 2 | 0.00 | 4 | 0.00 | 1 | 0.00 | 1 | 0.00 |  | 0.00 | 8 | 0.00 |
| W698 Drowning and submersion while in natural water, other specified places | 1 | 0.00 | 9 | 0.00 | 7 | 0.00 | 7 | 0.00 | 1 | 0.00 |  | 0.00 | 25 | 0.00 |
| W699 Drowning and submersion while in natural water, unspecified place | 16 | 0.00 | 67 | 0.01 | 38 | 0.01 | 73 | 0.04 | 10 | 0.00 | 5 | 0.00 | 209 | 0.01 |
| W70 Drowning and submersion following fall into natural water | 106 | 0.03 | 135 | 0.02 | 100 | 0.02 | 62 | 0.03 | 73 | 0.01 | 51 | 0.01 | 527 | 0.02 |
| W700 Drowning and submersion following fall into natural water, home | 19 | 0.01 | 18 | 0.00 | 26 | 0.00 | 44 | 0.02 | 28 | 0.01 | 34 | 0.01 | 169 | 0.01 |
| W701 Drowning and submersion following fall into natural water, residential | 1 | 0.00 | 1 | 0.00 | 2 | 0.00 |  | 0.00 |  | 0.00 |  | 0.00 | 4 | 0.00 |
| W702 Drowning and submersion following fall into natural water, school, other | 1 | 0.00 | 1 | 0.00 | 1 | 0.00 |  | 0.00 | 2 | 0.00 | 1 | 0.00 | 6 | 0.00 |
| W703 Drowning and submersion following fall into natural water, sports and |  | 0.00 | 2 | 0.00 |  | 0.00 |  | 0.00 |  | 0.00 |  | 0.00 | 2 | 0.00 |
| W704 Drowning and submersion following fall into natural water, street and | 3 | 0.00 | 2 | 0.00 | 4 | 0.00 | 1 | 0.00 | 1 | 0.00 |  | 0.00 | 11 | 0.00 |
| W705 Drowning and submersion following fall into natural water, trade and s |  | 0.00 |  | 0.00 |  | 0.00 | 1 | 0.00 |  | 0.00 | 1 | 0.00 | 2 | 0.00 |
| W706 Drowning and submersion following fall into natural water, industrial |  | 0.00 |  | 0.00 | 3 | 0.00 |  | 0.00 |  | 0.00 | 2 | 0.00 | 5 | 0.00 |
| W708 Drowning and submersion following fall into natural water, other specified |  | 0.00 |  | 0.00 |  | 0.00 | 1 | 0.00 | 1 | 0.00 |  | 0.00 | 2 | 0.00 |
| W709 Drowning and submersion following fall into natural water, unspecified |  | 0.00 | 4 | 0.00 | 7 | 0.00 | 1 | 0.00 | 1 | 0.00 | 1 | 0.00 | 14 | 0.00 |
| W74 Unspecified drowning and submersion | 34 | 0.01 | 42 | 0.01 | 35 | 0.01 | 21 | 0.01 | 31 | 0.01 | 65 | 0.01 | 228 | 0.01 |
| W740 Unspecified drowning and submersion, home | 5 | 0.00 | 5 | 0.00 | 4 | 0.00 | 11 | 0.01 | 14 | 0.00 | 26 | 0.00 | 65 | 0.00 |
| W741 Unspecified drowning and submersion, residential institution |  | 0.00 |  | 0.00 | 3 | 0.00 |  | 0.00 |  | 0.00 | 1 | 0.00 | 4 | 0.00 |
| W744 Unspecified drowning and submersion, street and highway | 2 | 0.00 | 2 | 0.00 |  | 0.00 |  | 0.00 |  | 0.00 | 1 | 0.00 | 5 | 0.00 |
| W745 Unspecified drowning and submersion, trade and service area | 1 | 0.00 |  | 0.00 | 1 | 0.00 |  | 0.00 |  | 0.00 | 2 | 0.00 | 4 | 0.00 |
| W746 Unspecified drowning and submersion, industrial and construction area |  | 0.00 |  | 0.00 |  | 0.00 |  | 0.00 |  | 0.00 | 1 | 0.00 | 1 | 0.00 |
| W747 Unspecified drowning and submersion, farm |  | 0.00 |  | 0.00 | 1 | 0.00 | 1 | 0.00 | 1 | 0.00 |  | 0.00 | 3 | 0.00 |
| W748 Unspecified drowning and submersion, other specified places |  | 0.00 |  | 0.00 |  | 0.00 |  | 0.00 |  | 0.00 | 20 | 0.00 | 20 | 0.00 |
| W749 Unspecified drowning and submersion, unspecified place | 4 | 0.00 | 9 | 0.00 | 2 | 0.00 |  | 0.00 | 6 | 0.00 | 11 | 0.00 | 32 | 0.00 |
| X20 Contact with venomous snakes and lizards | 82 | 0.02 | 132 | 0.02 | 324 | 0.05 | 246 | 0.12 | 281 | 0.05 | 555 | 0.09 | 1620 | 0.06 |
| X200 Contact with venomous snakes and lizards, home | 89 | 0.02 | 114 | 0.02 | 162 | 0.03 | 248 | 0.12 | 286 | 0.05 | 312 | 0.05 | 1211 | 0.04 |
| X201 Contact with venomous snakes and lizards, residential institution | 7 | 0.00 | 8 | 0.00 | 12 | 0.00 | 19 | 0.01 | 11 | 0.00 | 16 | 0.00 | 73 | 0.00 |
| X202 Contact with venomous snakes and lizards, school, other institution an | 4 | 0.00 | 7 | 0.00 | 3 | 0.00 | 3 | 0.00 | 1 | 0.00 | 6 | 0.00 | 24 | 0.00 |
| X203 Contact with venomous snakes and lizards, sports and athletics area | 3 | 0.00 | 10 | 0.00 | 40 | 0.01 | 40 | 0.02 | 3 | 0.00 | 3 | 0.00 | 99 | 0.00 |
| X204 Contact with venomous snakes and lizards, street and highway | 2 | 0.00 | 6 | 0.00 | 4 | 0.00 | 6 | 0.00 | 1 | 0.00 | 4 | 0.00 | 23 | 0.00 |
| X205 Contact with venomous snakes and lizards, trade and service area | 2 | 0.00 | 2 | 0.00 | 5 | 0.00 | 3 | 0.00 |  | 0.00 |  | 0.00 | 12 | 0.00 |
| X206 Contact with venomous snakes and lizards, industrial and construction | 4 | 0.00 |  | 0.00 | 2 | 0.00 | 2 | 0.00 |  | 0.00 |  | 0.00 | 8 | 0.00 |
| X207 Contact with venomous snakes and lizards, farm | 4 | 0.00 | 9 | 0.00 | 8 | 0.00 | 7 | 0.00 | 3 | 0.00 | 4 | 0.00 | 35 | 0.00 |
| X208 Contact with venomous snakes and lizards, other specified places | 3 | 0.00 | 7 | 0.00 | 6 | 0.00 | 10 | 0.00 | 9 | 0.00 | 6 | 0.00 | 41 | 0.00 |
| X209 Contact with venomous snakes and lizards, unspecified place | 49 | 0.01 | 42 | 0.01 | 40 | 0.01 | 32 | 0.02 | 23 | 0.00 | 39 | 0.01 | 225 | 0.01 |

**Supplementary table 14.** High prevalent climate sensitive diseases classifications

| **Disease classification** | **Sub-classification** |
| --- | --- |
| Cholera | A00 Cholera |
|  | A000 Cholera due to Vibrio cholerae 01, biovar cholerae |
|  | A001 Cholera due to Vibrio cholerae 01, biovar eltor |
|  | A009 Cholera, unspecified |
| Typhoid fever | A01 Typhoid and paratyphoid fevers |
|  | A010 Typhoid fever |
|  | A011 Paratyphoid fever A |
|  | A012 Paratyphoid fever B |
|  | A013 Paratyphoid fever C |
|  | A014 Paratyphoid fever, unspecified |
| Diarrhoea and gastroenteritis of presumed infectious origin | A09 Diarrhoea and gastroenteritis of presumed infectious origin |
|  | A090 Other and unspecified gastroenteritis and colitis of infectious origin |
| Dengue fever | A90 Dengue fever [classical dengue] |
|  | A91 Dengue haemorrhagic fever |
| Insulin-dependent diabetes mellitus | E10 Insulin-dependent diabetes mellitus |
|  | E100 Insulin-dependent diabetes mellitus with coma |
|  | E101 Insulin-dependent diabetes mellitus with ketoacidosis |
|  | E102 Insulin-dependent diabetes mellitus with renal complications |
|  | E103 Insulin-dependent diabetes mellitus with ophthalmic complications |
|  | E104 Insulin-dependent diabetes mellitus with neurological complications |
|  | E105 Insulin-dependent diabetes mellitus with peripheral circulatory compli |
|  | E106 Insulin-dependent diabetes mellitus with other specified complications |
|  | E107 Insulin-dependent diabetes mellitus with multiple complications |
|  | E108 Insulin-dependent diabetes mellitus with unspecified complications |
|  | E109 Insulin-dependent diabetes mellitus without complications |
| Non-insulin-dependent diabetes mellitus | E11 Non-insulin-dependent diabetes mellitus |
|  | E110 Non-insulin-dependent diabetes mellitus with coma |
|  | E111 Non-insulin-dependent diabetes mellitus with ketoacidosis |
|  | E112 Non-insulin-dependent diabetes mellitus with renal complications |
|  | E113 Non-insulin-dependent diabetes mellitus with ophthalmic complications |
|  | E114 Non-insulin-dependent diabetes mellitus with neurological complication |
|  | E115 Non-insulin-dependent diabetes mellitus with peripheral circulatory co |
|  | E116 Non-insulin-dependent diabetes mellitus with other specified complicat |
|  | E117 Non-insulin-dependent diabetes mellitus with multiple complications |
|  | E118 Non-insulin-dependent diabetes mellitus with unspecified complications |
|  | E119 Non-insulin-dependent diabetes mellitus without complications |
| Malnutrition-related and other specified or unspecified diabetes mellitus | E12 Malnutrition-related diabetes mellitus |
|  | E120 Malnutrition-related diabetes mellitus with coma |
|  | E121 Malnutrition-related diabetes mellitus with ketoacidosis |
|  | E122 Malnutrition-related diabetes mellitus with renal complications |
|  | E123 Malnutrition-related diabetes mellitus with ophthalmic complications |
|  | E124 Malnutrition-related diabetes mellitus with neurological complications |
|  | E125 Malnutrition-related diabetes mellitus with peripheral circulatory com |
|  | E126 Malnutrition-related diabetes mellitus with other specified complicati |
|  | E127 Malnutrition-related diabetes mellitus with multiple complications |
|  | E128 Malnutrition-related diabetes mellitus with unspecified complications |
|  | E129 Malnutrition-related diabetes mellitus without complications |
|  | E13 Other specified diabetes mellitus |
|  | E130 Other specified diabetes mellitus with coma |
|  | E131 Other specified diabetes mellitus with ketoacidosis |
|  | E132 Other specified diabetes mellitus with renal complications |
|  | E133 Other specified diabetes mellitus with ophthalmic complications |
|  | E134 Other specified diabetes mellitus with neurological complications |
|  | E135 Other specified diabetes mellitus with peripheral circulatory complica |
|  | E136 Other specified diabetes mellitus with other specified complications |
|  | E137 Other specified diabetes mellitus with multiple complications |
|  | E138 Other specified diabetes mellitus with unspecified complications |
|  | E139 Other specified diabetes mellitus without complications |
|  | E14 Unspecified diabetes mellitus |
|  | E140 Unspecified diabetes mellitus with coma |
|  | E141 Unspecified diabetes mellitus with ketoacidosis |
|  | E142 Unspecified diabetes mellitus with renal complications |
|  | E143 Unspecified diabetes mellitus with ophthalmic complications |
|  | E144 Unspecified diabetes mellitus with neurological complications |
|  | E145 Unspecified diabetes mellitus with peripheral circulatory complication |
|  | E146 Unspecified diabetes mellitus with other specified complications |
|  | E147 Unspecified diabetes mellitus with multiple complications |
|  | E148 Unspecified diabetes mellitus with unspecified complications |
|  | E149 Unspecified diabetes mellitus without complications |
| Phobic and other anxiety disorders | F40 Phobic anxiety disorders |
|  | F400 Agoraphobia |
|  | F401 Social phobias |
|  | F402 Specific (isolated) phobias |
|  | F408 Other phobic anxiety disorders |
|  | F409 Phobic anxiety disorder, unspecified |
| Anxiety disorder, panic, generalised and others. | F41 Other anxiety disorders |
|  | F410 Panic disorder [episodic paroxysmal anxiety] |
|  | F411 Generalized anxiety disorder |
|  | F412 Mixed anxiety and depressive disorder |
|  | F413 Other mixed anxiety disorders |
|  | F418 Other specified anxiety disorders |
|  | F419 Anxiety disorder, unspecified |
| Dissociative and Somatoform disorders | F44 Dissociative [conversion] disorders |
|  | F440 Dissociative amnesia |
|  | F441 Dissociative fugue |
|  | F442 Dissociative stupor |
|  | F443 Trance and possession disorders |
|  | F444 Dissociative motor disorders |
|  | F445 Dissociative convulsions |
|  | F446 Dissociative anaesthesia and sensory loss |
|  | F447 Mixed dissociative [conversion] disorders |
|  | F448 Other dissociative [conversion] disorders |
|  | F449 Dissociative [conversion] disorder, unspecified |
|  | F45 Somatoform disorders |
|  | F450 Somatization disorder |
|  | F451 Undifferentiated somatoform disorder |
|  | F452 Hypochondriacal disorder |
|  | F453 Somatoform autonomic dysfunction |
|  | F454 Persistent somatoform pain disorder |
|  | F458 Other somatoform disorders |
|  | F459 Somatoform disorder, unspecified |
| Viral pneumonia, NEC | J12 Viral pneumonia, NEC |
|  | J120 Adenoviral pneumonia |
|  | J121 Respiratory syncytial virus pneumonia |
|  | J122 Parainfluenza virus pneumonia |
|  | J123 Human metapneumovirus pneumonia |
|  | J128 Other viral pneumonia |
|  | J129 Viral pneumonia, unspecified |
| Bacterial pneumonia | J13 Pneumonia due to Streptococcus pneumoniae |
|  | J14 Pneumonia due to Haemophilus influenzae |
|  | J15 Bacterial pneumonia, NEC |
|  | J150 Pneumonia due to Klebsiella pneumoniae |
|  | J151 Pneumonia due to Pseudomonas |
|  | J152 Pneumonia due to staphylococcus |
|  | J153 Pneumonia due to streptococcus, group B |
|  | J154 Pneumonia due to other streptococci |
|  | J155 Pneumonia due to Escherichia coli |
|  | J156 Pneumonia due to other aerobic Gram-negative bacteria |

**Supplementary table 15.** Prevalence of major climate sensitive disease in Bangladesh, year 2017-2022

| **Diseases** | **n** | **%** |
| --- | --- | --- |
| Cholera | 86840 | 3.03 |
| Typhoid fever | 93668 | 3.27 |
| Diarrhoea and gastroenteritis of presumed infectious origin | 816908 | 28.51 |
| Dengue fever | 57123 | 1.99 |
| Insulin-dependent diabetes mellitus | 83800 | 2.92 |
| Non-insulin-dependent diabetes mellitus | 29758 | 1.04 |
| Malnutrition-related and other specifed or unspecified diabetes mellitus | 42802 | 1.49 |
| Phobic and other anxiety disorders | 12579 | 0.44 |
| Anxiety disorder, panic, generalised and others | 376814 | 13.15 |
| Dissociative and Somatoform disorders | 132011 | 4.61 |
| Viral pneumonia, NEC | 53935 | 1.88 |
| Bacterial pneumonia | 44938 | 1.57 |
| Other pneumonia | 541084 | 18.88 |
| Urinary tract infection | 225515 | 7.87 |
| Others | 267591 | 9.34 |
| Total | 2865366 | 100 |

**Supplementary table 16. District-wise distribution of climate sensitive diseases**

| **District** | **Cholera** | **Typhoid fever** | **Diarrhoea and gastroenteritis of presumed infectious origin** | **Dengue fever** | **Insulin-dependent diabetes mellitus** | **Non-insulin-dependent diabetes mellitus** | **Malnutrition-related and other specified or unspecified diabetes mellitus** | **Phobic and other anxiety disorders** | **Anxiety disorder, panic, generalised and others** | **Dissociative and Somatoform disorders** | **Viral pneumonia, NEC** | **Bacterial pneumonia** | **Other pneumonia** | **Urinary tract infection** | **others** | **Total** |
| --- | --- | --- | --- | --- | --- | --- | --- | --- | --- | --- | --- | --- | --- | --- | --- | --- |
| Bagerhat | 578 | 5646 | 13164 | 581 | 1359 | 657 | 252 | 89 | 3137 | 1454 | 544 | 532 | 1782 | 2668 | 2923 | 35366 |
| Bandarban | 40 | 1040 | 4051 | 681 | 94 | 115 | 74 | 22 | 195 | 413 | 868 | 348 | 2494 | 949 | 3933 | 15317 |
| Barguna | 385 | 81 | 9583 | 925 | 239 | 159 | 50 | 23 | 1464 | 2370 | 486 | 15 | 2202 | 378 | 3444 | 21804 |
| Barishal | 1215 | 2014 | 25870 | 666 | 248 | 217 | 379 | 38 | 347 | 1453 | 566 | 1568 | 5140 | 1104 | 4159 | 44984 |
| Bhola | 1241 | 754 | 15230 | 985 | 397 | 225 | 187 | 1403 | 1109 | 2056 | 303 | 3883 | 14581 | 2273 | 2485 | 47112 |
| Bogura | 108 | 1314 | 13434 | 1214 | 5803 | 5653 | 2646 | 665 | 8475 | 2128 | 1322 | 200 | 11969 | 7222 | 10749 | 72902 |
| Brahmanbaria | 1742 | 745 | 17566 | 787 | 1629 | 202 | 256 | 351 | 2478 | 1570 | 895 | 243 | 6033 | 1945 | 2147 | 38589 |
| Chandpur | 2063 | 2901 | 5370 | 2095 | 2022 | 298 | 618 | 103 | 3506 | 4686 | 3028 | 2033 | 9368 | 4387 | 3788 | 46266 |
| Chapainawabganj | 8721 | 629 | 9371 | 516 | 593 | 77 | 1175 | 48 | 6227 | 1035 | 325 | 44 | 12183 | 2941 | 1638 | 45523 |
| Chattogram | 2482 | 2289 | 42468 | 2402 | 1777 | 2691 | 1234 | 130 | 1137 | 1885 | 1108 | 1403 | 27876 | 4474 | 7369 | 100725 |
| Chuadanga | 36 | 1020 | 3558 | 54 | 1622 | 62 | 82 | 64 | 7846 | 231 | 923 | 27 | 743 | 754 | 1967 | 18989 |
| Cox's Bazar | 1295 | 157 | 13751 | 3195 | 1015 | 1413 | 345 | 412 | 4962 | 2522 | 439 | 2028 | 29992 | 5190 | 7884 | 74600 |
| Cumilla | 3263 | 2188 | 28531 | 707 | 1226 | 464 | 480 | 103 | 882 | 5873 | 1985 | 615 | 14088 | 4400 | 4495 | 69300 |
| Dhaka | 205 | 532 | 3015 | 5593 | 2876 | 350 | 696 | 109 | 919 | 504 | 2187 | 1417 | 4216 | 2911 | 12006 | 37536 |
| Dinajpur | 903 | 567 | 6235 | 135 | 2006 | 507 | 739 | 125 | 10478 | 888 | 1262 | 99 | 3999 | 4533 | 6749 | 39225 |
| Faridpur | 2933 | 1227 | 16887 | 2285 | 714 | 301 | 302 | 148 | 6820 | 4718 | 130 | 1536 | 2232 | 2895 | 9030 | 52158 |
| Feni | 1330 | 5707 | 19274 | 229 | 421 | 147 | 197 | 29 | 4637 | 1639 | 459 | 90 | 5409 | 1812 | 3404 | 44784 |
| Gaibandha | 7818 | 3642 | 28196 | 193 | 770 | 180 | 41 | 73 | 3386 | 1744 | 309 | 55 | 3287 | 1620 | 2521 | 53835 |
| Gazipur | 425 | 671 | 4556 | 1380 | 836 | 663 | 699 | 208 | 3064 | 2051 | 383 | 549 | 7360 | 6512 | 3332 | 32689 |
| Gopalganj | 2833 | 1034 | 9288 | 680 | 1079 | 483 | 142 | 51 | 5616 | 1588 | 446 | 387 | 2321 | 4445 | 3065 | 33458 |
| Habiganj | 917 | 102 | 11637 | 165 | 202 | 16 | 270 | 111 | 2687 | 3006 | 305 | 979 | 12432 | 3369 | 1691 | 37889 |
| Jamalpur | 1046 | 923 | 16517 | 554 | 1208 | 64 | 193 | 11 | 1418 | 799 | 155 | 69 | 2397 | 2017 | 3040 | 30411 |
| Jashore | 25 | 695 | 8048 | 4246 | 1949 | 127 | 4684 | 25 | 27817 | 7647 | 50 | 19 | 5777 | 13751 | 8143 | 83003 |
| Jhalokathi | 83 | 337 | 8028 | 296 | 471 | 193 | 167 | 142 | 3121 | 422 | 415 | 266 | 1708 | 1122 | 2001 | 18772 |
| Jhenaidah | 3006 | 2209 | 21019 | 707 | 4485 | 772 | 52 | 83 | 25349 | 5387 | 4817 | 1809 | 11117 | 6472 | 7415 | 94699 |
| Joypurhat | 42 | 1245 | 4327 | 86 | 1072 | 42 | 1153 | 89 | 5713 | 1327 | 286 | 29 | 4503 | 3706 | 1601 | 25221 |
| Khagrachari | 87 | 702 | 2440 | 349 | 849 | 210 | 408 | 62 | 773 | 1076 | 363 | 18 | 9224 | 2062 | 2096 | 20719 |
| Khulna | 308 | 452 | 5230 | 1166 | 4004 | 1772 | 1412 | 89 | 8182 | 3690 | 615 | 80 | 5730 | 3002 | 7170 | 42902 |
| Kishoreganj | 1008 | 2167 | 24874 | 1467 | 829 | 554 | 198 | 105 | 11892 | 2005 | 2194 | 821 | 15468 | 7048 | 8594 | 79224 |
| Kurigram | 1858 | 306 | 4782 | 354 | 2735 | 132 | 71 | 3073 | 5113 | 1406 | 999 | 48 | 1462 | 3280 | 3006 | 28625 |
| Kushtia | 22 | 1109 | 7308 | 1375 | 1459 | 319 | 327 | 213 | 12878 | 697 | 232 | 108 | 2791 | 3108 | 4153 | 36099 |
| Lakshmipur | 1143 | 764 | 21758 | 696 | 962 | 369 | 630 | 105 | 1042 | 1392 | 104 | 2330 | 10975 | 2479 | 6568 | 51317 |
| Lalmonirhat | 36 | 108 | 6314 | 125 | 503 | 140 | 62 | 99 | 1791 | 691 | 780 | 42 | 2065 | 884 | 1011 | 14651 |
| Madaripur | 1118 | 490 | 13753 | 1181 | 717 | 31 | 211 | 16 | 7191 | 2044 | 450 | 112 | 6467 | 1992 | 1769 | 37542 |
| Magura | 328 | 3495 | 3394 | 743 | 1185 | 43 | 309 | 159 | 4346 | 290 | 267 | 153 | 1132 | 1625 | 5050 | 22519 |
| Manikganj | 349 | 1897 | 4860 | 1281 | 754 | 153 | 227 | 158 | 928 | 784 | 887 | 257 | 1040 | 2193 | 1976 | 17744 |
| Maulvibazar | 343 | 177 | 12894 | 138 | 210 | 169 | 1132 | 75 | 6212 | 2486 | 419 | 275 | 14763 | 9319 | 3080 | 51692 |
| Meherpur | 311 | 211 | 6260 | 340 | 1966 | 155 | 526 | 75 | 10528 | 580 | 395 | 86 | 4630 | 4808 | 1229 | 32100 |
| Munshiganj | 163 | 323 | 12693 | 847 | 1205 | 676 | 149 | 111 | 3115 | 132 | 376 | 38 | 1955 | 3421 | 3432 | 28636 |
| Mymensingh | 480 | 1192 | 19813 | 467 | 1234 | 380 | 152 | 440 | 5250 | 5492 | 5286 | 3317 | 13070 | 5740 | 12887 | 75200 |
| Naogaon | 1686 | 1895 | 16992 | 234 | 2810 | 511 | 793 | 436 | 20075 | 1214 | 320 | 50 | 2577 | 3427 | 3825 | 56845 |
| Narail | 1368 | 7714 | 3029 | 884 | 595 | 285 | 17 | 19 | 810 | 471 | 829 | 9996 | 408 | 774 | 1633 | 28832 |
| Narayanganj | 61 | 1071 | 6573 | 1116 | 558 | 487 | 342 | 12 | 149 | 96 | 412 | 92 | 1566 | 1206 | 1422 | 15163 |
| Narsingdi | 99 | 1480 | 13580 | 876 | 485 | 494 | 315 | 37 | 848 | 746 | 213 | 207 | 8110 | 1831 | 1807 | 31128 |
| Natore | 64 | 324 | 12218 | 266 | 2343 | 290 | 1776 | 34 | 11734 | 1422 | 47 | 26 | 8163 | 3542 | 1721 | 43970 |
| Netrakona | 432 | 1258 | 25839 | 107 | 317 | 125 | 819 | 36 | 5197 | 3180 | 387 | 405 | 10077 | 3672 | 3002 | 54853 |
| Nilphamari | 6686 | 8780 | 13999 | 115 | 743 | 95 | 141 | 301 | 9188 | 1365 | 1683 | 96 | 4501 | 3092 | 4149 | 54934 |
| Noakhali | 918 | 327 | 6655 | 297 | 482 | 154 | 104 | 19 | 1034 | 1893 | 130 | 50 | 13829 | 1845 | 1814 | 29551 |
| Pabna | 4870 | 3156 | 30585 | 1373 | 1916 | 386 | 2065 | 87 | 22469 | 2188 | 370 | 84 | 15039 | 9035 | 6868 | 100491 |
| Panchagarh | 183 | 211 | 11420 | 55 | 229 | 165 | 596 | 36 | 9040 | 2136 | 545 | 22 | 1590 | 1644 | 1770 | 29642 |
| Patuakhali | 1704 | 1222 | 13248 | 828 | 391 | 914 | 197 | 36 | 408 | 4153 | 352 | 37 | 14233 | 1592 | 6000 | 45315 |
| Pirojpur | 95 | 544 | 11825 | 1498 | 316 | 16 | 915 | 13 | 2784 | 2319 | 21 | 16 | 9800 | 1939 | 2667 | 34768 |
| Rajbari | 18 | 95 | 5377 | 475 | 195 | 51 | 1304 | 42 | 5139 | 6220 | 165 | 52 | 4290 | 2474 | 1872 | 27769 |
| Rajshahi | 5818 | 7179 | 48968 | 1473 | 5709 | 686 | 3615 | 422 | 28213 | 3075 | 920 | 209 | 11358 | 16690 | 12953 | 147288 |
| Rangamati | 4 | 125 | 4444 | 233 | 850 | 263 | 649 | 6 | 818 | 470 | 116 | 79 | 1548 | 1364 | 2172 | 13141 |
| Rangpur | 1072 | 507 | 2273 | 33 | 1690 | 239 | 193 | 135 | 4367 | 1227 | 1558 | 1306 | 2829 | 2882 | 2588 | 22899 |
| Satkhira | 160 | 130 | 8339 | 818 | 1871 | 329 | 1104 | 43 | 3203 | 2505 | 774 | 522 | 4338 | 1079 | 1849 | 27064 |
| Shariatpur | 345 | 534 | 10284 | 988 | 221 | 32 | 326 | 172 | 615 | 2336 | 368 | 149 | 3255 | 1023 | 1088 | 21736 |
| Sherpur | 1368 | 440 | 6269 | 354 | 643 | 111 | 1487 | 98 | 2180 | 420 | 402 | 60 | 2822 | 3351 | 1734 | 21739 |
| Sirajganj | 3355 | 588 | 5607 | 929 | 838 | 170 | 627 | 113 | 3299 | 353 | 1546 | 105 | 4839 | 1261 | 7672 | 31302 |
| Sunamganj | 580 | 656 | 13444 | 67 | 414 | 67 | 202 | 644 | 1169 | 2455 | 378 | 178 | 38043 | 2980 | 2514 | 63791 |
| Sylhet | 2424 | 687 | 17108 | 29 | 1696 | 425 | 200 | 89 | 4569 | 1585 | 870 | 2028 | 29796 | 3525 | 3174 | 68205 |
| Tangail | 1144 | 653 | 11341 | 670 | 2593 | 405 | 696 | 199 | 5213 | 7607 | 650 | 1195 | 6320 | 4629 | 4276 | 47591 |
| Thakurgaon | 90 | 841 | 11874 | 211 | 289 | 32 | 1380 | 114 | 10986 | 275 | 74 | 33 | 37296 | 2451 | 3126 | 69072 |
| Total | 86833 | 93479 | 816638 | 55815 | 82919 | 27913 | 42790 | 12578 | 375538 | 131872 | 50493 | 44925 | 532608 | 222119 | 264696 | 2841216 |
