# Supplementary material for: Understanding climate-sensitive diseases in Bangladesh using systematic review and government data repository
Source: PLoS One. 2025 Mar 19;20(3):e0313031. doi: 10.1371/journal.pone.0313031 (PMC11922245; doi:10.1371/journal.pone.0313031)
Supplement: Attachment — Submitted filename: My comment.docx [file pone.0313031.s003.docx]

Based on the abstract and other sections I have read, here is my overall assessment of the paper along with some specific comments:

**Strengths:**

1. The study addresses an important gap in understanding the full range of climate-sensitive diseases occurring in Bangladesh, by comparing evidence from literature to government data records. This helps reveal discrepancies between current knowledge and actual reported cases.
2. A very comprehensive, systematic search strategy was used spanning multiple databases to identify relevant studies on climate change impacts on health in Bangladesh. This ensures a broad capture of existing evidence.
3. Analyzing a large dataset of over 2.8 million records from government system allows for examination of reported cases at scale. Breaking this down by disease types and years provides helpful insights.
4. Discussion rightly points out limitations around lack of comprehensive patient data as well as absence of unique identifiers in government records which poses challenges. Highlights the need for better quality data to inform policies and programs.

**Weaknesses:**

1. More details could have been provided on the exact search terms/syntax used in literature review databases to enable replicability. The current supplementary tables do not seem to capture full search strategies.
2. For government data, the quality control and validation measures for data collection/recording could have been elaborately discussed to instill more confidence in findings.
3. The results try to provide a year-wise breakdown of climate sensitive diseases but trends over time are not clearly analyzed through some visual plots or statistical tests. This could have brought out insights.
4. Conclusion seems to predominantly focus on data limitations. Could have provided some specific recommendations on how surveillance and reporting for climate sensitive diseases can be strengthened.

**Additional major comments:**

1. The rationale for choice of time period for literature review starting from 2000 needs to be justified.

2. Were there any quality assessment criteria set for including studies in review? This is important to mention.

3. Supplementary Table 13 shows a list of climate-sensitive diseases but it does not match with Figure 3 classifications. Needs consistency.

4. For government data, the sampling framework should be clearly described - how were the 516 facilities selected? Any region wise stratification done? This has implications for results.
