# Supplementary material for: Understanding climate-sensitive diseases in Bangladesh using systematic review and government data repository
Source: PLoS One. 2025 Mar 19;20(3):e0313031. doi: 10.1371/journal.pone.0313031 (PMC11922245; doi:10.1371/journal.pone.0313031)
Supplement: Attachment — Submitted filename: 2-Comment-2024-FM-UHS.docx [file pone.0313031.s005.docx]

**Addressing Data Challenges for Understanding Climate-Sensitive Diseases in Bangladesh: Evidence from Systematic Review and Government Data Repository**

The topic is very interesting, and deserve to be studied. However, there are some problems the authors should make them clear before accepting it for publication. The authors please take the following into account.

- The abstract provides a well-organized overview of the study, highlighting the critical issue of understanding climate change-related health outcomes in Bangladesh. While the key components of the research, such as the systematic review and comparison with government data, are effectively communicated, a bit more detail on the specific climate-sensitive diseases and potential implications for policy formulation would enhance the clarity and impact of the abstract. Additionally, ensuring consistency in terminology and briefly explaining key concepts for readers less familiar with the field would contribute to better overall comprehension.
- Consider ensuring a smooth transition between paragraphs, providing a seamless flow of information from the global context to Bangladesh's specific challenges.
- It would be beneficial to briefly list or elaborate on the specific climate-sensitive diseases mentioned in the context of Bangladesh, providing readers with a clearer understanding of the health risks.
- While the mention of the government's Climate Change Strategy and Action Plan is crucial, consider providing a bit more detail on specific measures or strategies outlined in the plan, highlighting how it addresses the health impact of climate change.
- Given that the search was initially conducted in December 2022 and later updated in July 2023, briefly discuss any potential reasons for the update and how it contributed to the study's comprehensiveness.
- Briefly discuss the rationale behind choosing narrative synthesis as the method for summarizing findings, and highlight any key themes or patterns that emerged during this synthesis.
- Author need to check some typesetting errors throughout the manuscript.
- Discuss in more detail the challenges associated with the current data collection through DHIS 2. Highlight specific issues related to patient demographics, community characteristics, and the lack of unique identification numbers.
- Elaborate on the identified limitations of the government-recorded data, emphasizing their impact on policymakers and program developers. Discuss how these limitations may hinder the effective management of climate-sensitive diseases.
- Reinforce the discussion's connection to the study's objectives. Explicitly state how each finding contributes to achieving the research goals, providing a clear linkage between the results and the initial study objectives.
