# Supplementary material for: Understanding climate-sensitive diseases in Bangladesh using systematic review and government data repository
Source: PLoS One. 2025 Mar 19;20(3):e0313031. doi: 10.1371/journal.pone.0313031 (PMC11922245; doi:10.1371/journal.pone.0313031)
Supplement: Attachment — Submitted filename: Response to reviewers.docx [file pone.0313031.s006.docx]

**Authors’ responses to reviewers’ comments**

| **Reviewers’ comment** | **Authors’ response** |
| --- | --- |
| **Reviewer#1** |  |
| **Strengths** |  |
| 1. The study addresses an important gap in understanding the full range of climate-sensitive diseases occurring in Bangladesh, by comparing evidence from literature to government data records. This helps reveal discrepancies between current knowledge and actual reported cases. | Thank you for recognizing the importance of our study in addressing the gap in understanding climate-sensitive diseases in Bangladesh. |
| 2. A very comprehensive, systematic search strategy was used spanning multiple databases to identify relevant studies on climate change impacts on health in Bangladesh. This ensures a broad capture of existing evidence. | Thank you. No change is required to address this general observation. |
| 3. Analyzing a large dataset of over 2.8 million records from government system allows for examination of reported cases at scale. Breaking this down by disease types and years provides helpful insights. | Thank you. No change is required to address this general observation. |
| 4. Discussion rightly points out limitations around lack of comprehensive patient data as well as absence of unique identifiers in government records which poses challenges. Highlights the need for better quality data to inform policies and programs. | Thank you. No change is required to address this general observation. |
| **Weaknesses** |  |
| 1. More details could have been provided on the exact search terms/syntax used in literature review databases to enable replicability. The current supplementary tables do not seem to capture full search strategies. | We have now added the search strategy for each of the six databases in the Supplementary file. Please refer to Supplementary Tables 1-6. During the database search, broad terms were considered instead of specific search terms to include a broad range of papers from Bangladesh. |
| 2. For government data, the quality control and validation measures for data collection/recording could have been elaborately discussed to instill more confidence in findings. | We have now added the following sentences in the Exploration of respiratory health data from governmental database section of the methods to address your comment-  *The data was recorded under the supervision of the Ministry of Health and Welfare of Bangladesh. They have a separate unit called the Management Information System (MIS) to record this information along with other relevant data. They also conducted regular monitoring visits to ensure data accuracy. At the field level, they have expert personnel to record data.*  We also have the following sentences in the strengths and limitations section that present strength of the quantitative data we analysed-  *Strict quality control measures ensured while collecting this data. Additionally, the utilization of the ICD-10 criteria for classifying these diseases and the investigation of the most prevalent conditions provides valuable insights for policymakers.* |
| 3. The results try to provide a year-wise breakdown of climate sensitive diseases but trends over time are not clearly analyzed through some visual plots or statistical tests. This could have brought out insights. | We did not find any specific trend in climate-sensitive diseases over the years. Therefore, we did not present it broadly as we already have a higher number of tables and figures. However, we have now clarified this issue of no specific trend in the relevant section of the methods by adding the following sentences:  *The distribution of these more prevalent diseases was examined on a yearly basis, and the findings are presented in Table 4. We did not find any specific trend of climate-sensitive diseases, with some years showing a notable increase that subsequently declined in the following years. In general, the prevalence of most of these diseases showed an increase in 2019, except for cholera, which exhibited an increase in 2017.* |
| 4. Conclusion seems to predominantly focus on data limitations. Could have provided some specific recommendations on how surveillance and reporting for climate sensitive diseases can be strengthened. | We have now added the following sentences in the conclusion section of the manuscript to address your comment-  *Improving the digital information management system, establishing a centralized database with unique patient identifiers, and providing training for healthcare professionals are essential steps. Integrating data from meteorological agencies into surveillance systems ensures specificity and facilitates the formulation of relevant policies and programs.* |
| 5. The rationale for choice of time period for literature review starting from 2000 needs to be justified. | We have selected the year 2000 as the starting point due to the initiation of the Millennium Development Goals. We have updated the inclusion criteria accordingly. It now states:  *Published from January 2000 to July 2023, aligning with the beginning of the Millennium Development Goals in January 2000.* |
| 6. Were there any quality assessment criteria set for including studies in review? This is important to mention. | We have considered the well-recognised Newcastle-Ottawa scale to measure the quality of the included studies. The relevant results are presented in the Supplementary table 7-11. The relevant results are discussed in the quality assessment of the included studies section of the results. It reads-  *The quality assessment of the included studies was conducted using the modified Newcastle-Ottawa Scale [16]. The scale encompassed specific criteria for cross-sectional (n=26), case-control (n=2), cohort studies (n=2), qualitative studies (n=3), and Randomized Control Trial (n=1). Authors reviewed the articles and marked an “*” for each criterion met. The scores were then summed up and categorized into three groups: high quality study (if the study achieved over 75% of the total allocated score), moderate quality (if the study achieved 50 to 74% of the total allocated score), and low quality (if the study achieved below 50% of the total allocated score). The majority of the studies were of moderate quality (n=27), followed by low quality (n=6) and only one high-quality study (n=1) (Supplementary table 7-11).* |
| 7. Supplementary Table 13 shows a list of climate-sensitive diseases but it does not match with Figure 3 classifications. Needs consistency. | Table 13 displays the comprehensive list of climate-sensitive diseases based on the ICD-10 guidelines, totaling approximately 600 diseases. Due to the extensive nature of this list, it is impractical to include all diseases in a single figure. As a result, we have categorized them into major groups, as outlined in supplementary table 14, and provided relevant estimates in figure 3 of the manuscript. Additionally, we have updated figure 3 to include several new categories to enhance the presentation of information. |
| 8. For government data, the sampling framework should be clearly described - how were the 516 facilities selected? Any region wise stratification done? This has implications for results | The 516 healthcare facilities were selected for data collection based on their infrastructure capabilities. This has been clarified in the "Exploration of respiratory health data from governmental database" section of the methods.  ***………*** *The data was recorded under the supervision of the Ministry of Health and Welfare of Bangladesh. They have a separate unit called the Management Information System (MIS) to record this information along with other relevant data. They also conducted regular monitoring visits to ensure data accuracy. At the field level, they have expert personnel to record data. The data collection is currently being conducted at 516 healthcare facilities across the country, selected based on their infrastructural capabilities to record real-time data……….*  However, we acknowledge that the inclusion of only 516 hospitals may be a significant limitation of the study. Therefore, we have added the following sentences in the strengths and limitations section of the discussion to acknowledge this concern:  *Quantitative data was collected from 516 healthcare facilities, indicating that data from other healthcare facilities was not recorded. This represents a partial dataset that we analyzed. However, the broader geographical coverage of the hospitals from which data was collected suggests that the results are nationally representative.* |
| **Reviewer#2** |  |
| Thanks for your contribution, in short you can modify the title, result part need to include data, analysis need to in good shape like regression analysis, need to add few more figures, discussion part not that much attractive so there is opportunity to work on it and finally a lucrative conclusion along with recommendation is needed. | Many thanks for your comment. We have now edited the title as follows to address your comment-  *Understanding Climate-Sensitive Diseases in Bangladesh using Systematic Review and Government Data Repository*  We have included data in the result section of the abstract to address your second point. This edited section now reads-  *The available literature in Bangladesh reports only a few specific climate-sensitive diseases, including Diarrhea, Dengue, Cholera, Malaria, Pneumonia, Cardiovascular Diseases, Hypertension, Urinary-Tract Infections, and Malnutrition, which were also considered in few studies. This represents a segment of the total 510 reported climate-sensitive diseases in Bangladesh, of which 143 diseases were responsible for 90.66% of the total occurrences. The most common forms of diseases were diarrhea and gastroenteritis of presumed infectious (28.51%), pneumonia (18.88%), anxiety disorders, panic disorders, generalized anxiety disorders (13.2%), and others (13.15%). Additionally, urinary tract infections (7.87%), cholera (3.03%), and typhoid fever (3.27%) were other frequently reported climate-sensitive diseases. We also explored several challenges related to available data in the government repository, which include inadequate collection of patients’ comprehensive socio-demographic information and the absence of a unique patient identifier.*  The data we analyzed may deter us from using regression analysis, although we initially intended to do so. Furthermore, we have revised the Results and Discussion section to enhance comprehensiveness. Please refer to the updated version of the manuscript. |
| **Reviewer#3** |  |
| Addressing Data Challenges for Understanding Climate-Sensitive Diseases in Bangladesh: Evidence from Systematic Review and Government Data Repository.  The topic is very interesting, and deserve to be studied. However, there are some problems the authors should make them clear before accepting it for publication. The authors please take the following into account. | Many thanks for your time to minutely read our paper. We have addressed your comments and provided a point-by-point response below- |
| The abstract provides a well-organized overview of the study, highlighting the critical issue of understanding climate change-related health outcomes in Bangladesh. While the key components of the research, such as the systematic review and comparison with government data, are effectively communicated, a bit more detail on the specific climate-sensitive diseases and potential implications for policy formulation would enhance the clarity and impact of the abstract. Additionally, ensuring consistency in terminology and briefly explaining key concepts for readers less familiar with the field would contribute to better overall comprehension. | As part of addressing your comment and one of the comments from the first reviewer, we have made broad edits to the abstract section. Please review the revised abstract of the manuscript. |
| Consider ensuring a smooth transition between paragraphs, providing a seamless flow of information from the global context to Bangladesh’s specific challenges. | We have revised the relevant sections throughout the manuscript to address your comment. Please check the revised version of the manuscript. |
| It would be beneficial to briefly list or elaborate on the specific climate-sensitive diseases mentioned in the context of Bangladesh, providing readers with a clearer understanding of the health risks. | We have detailed out specific climate-sensitive diseases for Bangladesh in Supplementary Table 13. Please have a look. |
| While the mention of the government’s Climate Change Strategy and Action Plan is crucial, consider providing a bit more detail on specific measures or strategies outlined in the plan, highlighting how it addresses the health impact of climate change. | We have now edited the relevant section of the introduction section to presrnt this. The edited relevant paragraph now reads-  *The Government of Bangladesh formulated a Climate Change Strategy and Action Plan in 2008, later updated in 2009, to tackle climate-sensitive diseases [11]. The plan aims to comprehensively assess the prevalence of climate-sensitive diseases across the country, considering geographical variations, and implement targeted policies and programs accordingly. However, this effort is impeded by the predominant focus on specific diseases such as diarrhea, dengue, cholera, malaria, pneumonia, cardiovascular diseases, hypertension, urinary-tract infections, and malnutrition in existing evidence. Establishing a surveillance system to monitor both existing and emerging climate-related diseases and strengthening health system resilience for the future are top priorities. Nevertheless, the effectiveness of these initiatives is hampered primarily by the lack of pertinent data. Currently available data mostly originates from small-scale regional studies, which often focus on a few specific outcomes and yield conflicting findings [12-15]. Although the government-initiated data collection and utilization efforts through District Health Information System 2 (DHIS 2) in 2009, the usability and coverage of this data remain largely unexplored. As a result of these complexities, there are limited knowledge regarding the climate change-related health outcomes in Bangladesh as compared to the global perspective, and the true extent of climate-sensitive diseases in the country remains unknown. This hampers the development of effective policies and programs to address the health impacts of climate change. To overcome this limitation, we conducted a mixed-methods study to explore the climate-sensitive diseases reported in existing literature in Bangladesh and compared them with the reported scenarios of relevant data in the government data repository. Furthermore, we examined the challenges associated with reporting climate change and related diseases data in the government data repository.* |
| Given that the search was initially conducted in December 2022 and later updated in July 2023, briefly discuss any potential reasons for the update and how it contributed to the study’s comprehensiveness. | This update aimed to include any additional papers published since the earlier search. We have now clarified this issue by editing the relevant sentences of the manuscript.  *We conducted a systematic literature search initially in December 2022 and later updated it in July 2023 to include any additional papers published since the initial search.* |
| Briefly discuss the rationale behind choosing narrative synthesis as the method for summarizing findings, and highlight any key themes or patterns that emerged during this synthesis. | The heterogeneity of the included studies in the systematic review prevented quantitative data analysis. Thus, we utilized narrative synthesis to summarize the findings. Additionally, we did not identify specific themes for consideration and broad discussion. This is also beyond the scope of our study, as our primary aim with the systematic review was to determine the extent and types of climate-sensitive diseases documented in the existing literature of Bangladesh. |
| Author need to check some typesetting errors throughout the manuscript. | We have meticulously reviewed the English language throughout the manuscript and corrected any errors. |
| Discuss in more detail the challenges associated with the current data collection through DHIS 2. Highlight specific issues related to patient demographics, community characteristics, and the lack of unique identification numbers. | We have thoroughly presented and discussed these points in the sixth and seventh paragraphs of the discussion section, which we have further refined to enhance clarity and precision in response to your feedback. Please refer to the revised version of the manuscript for details. |
| Elaborate on the identified limitations of the government-recorded data, emphasizing their impact on policymakers and program developers. Discuss how these limitations may hinder the effective management of climate-sensitive diseases. | We have now revised relevant section to address your comment. The relevant sentences now read-  *However, an important limitation of this study is the inability to provide summarized findings due to the inconsistent nature of the available literature. Quantitative data was recorded from 516 healthcare facilities indicating other healthcare facilities data was not recorded. This indicates a report of partial data that we analysed. However, broader geographical coverage of the hospitals from where data was recorded indicate the results are nationally representative. Moreover, the absence of unique patient identification numbers within the dataset poses challenges in distinguishing individuals reported multiple times across various healthcare facilities. Additionally, the lack of relevant data hampers the ability to assess the likelihood of disease occurrence based on respondents' characteristics. The partial data analysis underscores the challenge in formulating comprehensive policies and programs without a holistic understanding of healthcare trends. Without robust data collection methods and unique patient identifiers, policymakers may struggle to tailor interventions effectively to address emerging health issues………………….* |
| Reinforce the discussion’s connection to the study’s objectives. Explicitly state how each finding contributes to achieving the research goals, providing a clear linkage between the results and the initial study objectives. | We have revised the discussion section as necessary to ensure alignment with the study objectives. Please see the revised version of the manuscript. |
| **Reviewer#4** |  |
| Authors conduct a research on “Addressing Data Challenges for Understanding Climate-Sensitive Diseases in Bangladesh: Evidence from Systematic Review and Government Data Repository”. The present manuscript can be accepted for publication in Plos One after addressing the following minor issues. | Thank you for carefully reviewing our paper. We have addressed the comments you provided and have provided detailed responses below. |
| 1. Please reframe the abstract part, current abstract part is not attractive for readers. | We have revised the manuscript's abstract to address your feedback, as well as the comments from the other reviewers. Please see revised abstract in the manuscript. |
| 2. Please fine tuning the key words | We have rewritten the keywords as follows to address your comment-  *Climate-sensitive disease, District health information system 2, Government data respiratory, Systematic review, Bangladesh.* |
| 3. Modified the conclusion part for attraction of readers. | We have revised the conclusion section in both the abstract and body of the manuscript to address your comment. Please see the revised version of the manuscript. |
| 4. Update the all references | We have revised all references as per the PLoS ONE guideline of reference. Please see the revised version of the manuscript. |
| **Reviewer#5** |  |
| Very nice work on an important issue. I would suggest staying away from bombastic articles from the internet when citing impact of the climate changes (as cited on the page 3, reference 7). Good luck. | Many thanks for your time to read our paper. |
