# Supplementary material for: Understanding climate-sensitive diseases in Bangladesh using systematic review and government data repository
Source: PLoS One. 2025 Mar 19;20(3):e0313031. doi: 10.1371/journal.pone.0313031 (PMC11922245; doi:10.1371/journal.pone.0313031)
Supplement: Attachment — Submitted filename: Response_to_reviewers_auresp_2.docx [file pone.0313031.s007.docx]

**Authors’ responses to reviewers’ comments**

| **Reviewers’ comment** | **Authors’ response** |
| --- | --- |
| **Reviewer#2** |  |
| Please give a look again if any major mistake. Climate and its affect is a sensitive issues globally, before final task check it very carefully. | Thanks for your time to review our paper again. We have carefully look the entire manuscript again. |
| **Reviewer#6** |  |
| 1. Nothing was explained about the keyword "District health information system 2" in the abstract section. | Many thanks for your time to review our paper again.  This is a data collection process used by the government of Bangladesh to record hospital admission data. We have now deleted this keyword from the keywords section. |
| 2. Is the word "Government data respiratory" correct or should it be "Government data repository" ? Please check throughout the manuscript. | Many thanks to bring this unnoticed error in our attention. We have corrected this issue throughout the manuscript. |
| 3. Consistency can be maintained with full form and abbreviations. For example no abbreviation is given for Sustainable Development Goals. | The term Sustainable Development Goals was used only once in a manuscript. We therefore did not abbreviate the term. However, we have checked the entire manuscript carefully and fix this issue. |
| 4. The keywords that were used for systematic review can be shown in italic format. | We have now used italic front for the keywords used for systematic review to address your comment. |
| 5. Please check the title "Exploration of respiratory health data from governmental database" with the word respiratory. | We have fixed this issue. It is now reads: **Exploration of repository health data from governmental database** |
| 6. The line in the PRISMA description "Ultimately, 70 articles were included in this review, of which 36 were discarded reviewing the study design and finally 34 studies were included in qualitative synthesis. " is somewhat does not match with the diagram. Another box need be added in the diagram where the 70 articles can be added and then 36 exclusion can be shown. | Thanks to bring this unnoticed error in our attention. We have now edited the relevant sentences in the study selection section of the results section. The relevant sentences now read-  *A total of 1420 papers were initially identified through a comprehensive search across six databases, supplemented by additional identification of 11 papers via Google, Google Scholar, and a review of reference lists from the selected articles. After removal of duplicate entries, a refined list of 1367 unique articles was obtained. Upon screening of titles and abstracts based on predefined inclusion and exclusion criteria, 886 articles were excluded, as depicted in Figure 1. This left 315 articles for full-text review, among them 281 articles were excluded because of no outcome (n=245) and wrong study design (n=36). Finally 34 studies were included in qualitative synthesis.* |
| 7. By following the standard approach of PRISMA diagram, "identification", "screening" and "included" terms need to be added at the left side of the PRISMA diagram. | We have now your suggested terms with the PRISMA diagram to address your comment. |
| 8. Please check the typos such as along wise (along with), Randomized control trial (randomized control trial). | We have read the entre manuscript again and fixed the typos. |
| 9. Some terms need further clarification as it can be hard to understand what they mean. e.g. DALY. | We have now defined DAYs when it is first used. The relevant sentence now reads-  *Furthermore, climate-sensitive diseases contribute significantly to the loss of Disability Adjusted Life Years (DALYs, years of life lost due to premature mortality and years lived with disability or illness) among children.*  We also check the entire manuscript again and fixed all relevant issues. |
| 10. No full form is given for MoHFW. | We have now used the full term of MoHFW when it is first used. |
