# Supplementary material for: Understanding climate-sensitive diseases in Bangladesh using systematic review and government data repository
Source: PLoS One. 2025 Mar 19;20(3):e0313031. doi: 10.1371/journal.pone.0313031 (PMC11922245; doi:10.1371/journal.pone.0313031)
Supplement: Attachment — Submitted filename: Response to journal office comment after acceptence.docx [file pone.0313031.s008.docx]

**Acceptance email:**

CC: "Dewan Mashrur Hossain" mashrur.dewan@gmail.com, "Md. Toufiq Hassan Shawon" sawontheboss4@gmail.com, "Md. Mostaured Ali Khan" mostaured.khan@gmail.com, "Md Saiful Islam" dusaifstat64@gmail.com, "As Saba Hossain" as.saba.hossain@gmail.com, "Md. Nuruzzaman Khan" mdnuruzzaman.khan@uon.edu.au

Understanding Climate-Sensitive Diseases in Bangladesh using Systematic Review and Government Data Repository

PONE-D-23-35790R2

Dear Dr. Kabir,

We’re pleased to inform you that your manuscript has been judged scientifically suitable for publication and will be formally accepted for publication once it meets all outstanding technical requirements.

Within one week, you’ll receive an e-mail detailing the required amendments. When these have been addressed, you’ll receive a formal acceptance letter and your manuscript will be scheduled for publication.

An invoice will be generated when your article is formally accepted. Please note, if your institution has a publishing partnership with PLOS and your article meets the relevant criteria, all or part of your publication costs will be covered. Please make sure your user information is up-to-date by logging into Editorial Manager at [Editorial Manager®](https://url.au.m.mimecastprotect.com/s/2UwvC0YKM6TGZz1kpuwfLF9WrC6?domain=c05y1x9s.r.us-east-2.awstrack.me) and clicking the ‘Update My Information' link at the top of the page. If you have any questions relating to publication charges, please contact our Author Billing department directly at authorbilling@plos.org.

If your institution or institutions have a press office, please notify them about your upcoming paper to help maximize its impact. If they’ll be preparing press materials, please inform our press team as soon as possible -- no later than 48 hours after receiving the formal acceptance. Your manuscript will remain under strict press embargo until 2 pm Eastern Time on the date of publication. For more information, please contact onepress@plos.org.

Kind regards,

Md Jamal Uddin, Ph.D

Academic Editor

PLOS ONE

**Authors response: Thanks. No change is asked.**

**Follow-up email from journal office with requirement for additional information:**

PONE-D-23-35790R2

Understanding Climate-Sensitive Diseases in Bangladesh using Systematic Review and Government Data Repository

Dear Dr. Kabir:

Thank you for returning your manuscript and for your continued cooperation with our requests.  There are still some outstanding issues that must be resolved before your manuscript can be formally accepted – please see below.

To access your manuscript and complete these changes, please follow this link: <https://www.editorialmanager.com/pone/l.asp?i=67308412&l=W6NX2RQE>.

You will find the submission in “Current Task Assignments” and will need to click “Submit Task” in order to upload your corrected files. Your current files can be downloaded through the “Assignment Files” link; you can also download the manuscript file attached to this email.

Your task is due Nov 01 2024 11:59PM.

We appreciate your patience during this process.  Please contact [plosone@plos.org](mailto:plosone@plos.org) with any questions or concerns.

With kind regards,

PLOS ONE staff

JOURNAL REQUIREMENTS:

**"As required by our policy on Data Availability, please ensure your supplementary materials include a table of all studies identified in the literature search, with the full title of each article, including those excluded from the analyses. Please include the reasons for excluding these articles from your paper.**

Please note that if this is not provided, we may need to Reject your submission."

**Author’s response:** Please refer to Supplementary Table 17, which was uploaded separately due to its size. A summary of this information is already provided in Supplementary Tables 1 to 6.

Unfortunately, we inadvertently failed to attach the updated supplementary file, which may have contributed to the rejection of this paper after its preliminary acceptance. We hope this issue can be reconsidered, as such mistakes—uploading an incorrect file through email or an online submission system—can happen to anyone.

**Journal took decision to reject paper as the data was not supplied:**

PONE-D-23-35790R2
Understanding Climate-Sensitive Diseases in Bangladesh using Systematic Review and Government Data Repository
PLOS ONE

Dear Dr. Kabir,

Thank you for submitting your revised manuscript to PLOS ONE, and for responding to our recent requests regarding your submission. In our editorial checks of the documents that you supplied, we have concluded that your submission does not comply with our policies around data availability. We are therefore rejecting this manuscript.

PLOS journals require authors to make all data necessary to replicate their study’s findings publicly available without restriction at the time of publication (<https://journals.plos.org/plosone/s/data-availability>). In this case, the following underlying data were not provided as requested:

A numbered table of all studies identified in the literature search, including those that were excluded from the analyses.

As a result of these concerns, we cannot consider the manuscript for publication. I am very sorry that we cannot be more positive on this occasion.

Kind regards,

Jennifer Tucker, PhD
Staff Editor
PLOS ONE

**Author’s response:** As mentioned in our earlier email, we inadvertently failed to attach the updated supplementary file, which may have contributed to the rejection of the paper after its preliminary acceptance. However, we have now attached the missing data file, Supplementary Table 17. Accordingly, we are submitting an appeal, which has been approved, and the review process has been reinitiated.
